# Genome of the long-living sacred lotus (*Nelumbo nucifera *Gaertn.)

**DOI:** 10.1186/gb-2013-14-5-r41

**Published:** 2013-05-10

**Authors:** Ray Ming, Robert VanBuren, Yanling Liu, Mei Yang, Yuepeng Han, Lei-Ting Li, Qiong Zhang, Min-Jeong Kim, Michael C Schatz, Michael Campbell, Jingping Li, John E Bowers, Haibao Tang, Eric Lyons, Ann A Ferguson, Giuseppe Narzisi, David R Nelson, Crysten E Blaby-Haas, Andrea R Gschwend, Yuannian Jiao, Joshua P Der, Fanchang Zeng, Jennifer Han, Xiang Jia Min, Karen A Hudson, Ratnesh Singh, Aleel K Grennan, Steven J Karpowicz, Jennifer R Watling, Kikukatsu Ito, Sharon A Robinson, Matthew E Hudson, Qingyi Yu, Todd C Mockler, Andrew Carroll, Yun Zheng, Ramanjulu Sunkar, Ruizong Jia, Nancy Chen, Jie Arro, Ching Man Wai, Eric Wafula, Ashley Spence, Yanni Han, Liming Xu, Jisen Zhang, Rhiannon Peery, Miranda J Haus, Wenwei Xiong, James A Walsh, Jun Wu, Ming-Li Wang, Yun J Zhu, Robert E Paull, Anne B Britt, Chunguang Du, Stephen R Downie, Mary A Schuler, Todd P Michael, Steve P Long, Donald R Ort, J William Schopf, David R Gang, Ning Jiang, Mark Yandell, Claude W dePamphilis, Sabeeha S Merchant, Andrew H Paterson, Bob B Buchanan, Shaohua Li, Jane Shen-Miller

**Affiliations:** 1 Key Laboratory of Plant Germplasm Enhancement and Specialty Agriculture, Wuhan Botanical Garden, The Chinese Academy of Sciences, Lumo Road, Wuhan 430074, China; 2 Department of Plant Biology, University of Illinois at Urbana-Champaign, 1201 West Gregory Drive, Urbana, IL 61801, USA; 3 College of Horticulture, Nanjing Agricultural University, 1 Weigang Road, Nanjing 210095, China; 4 Institute of Biological Chemistry, Washington State University, Clark Hall, 100 Dairy Road, Pullman, WA 99164, USA; 5 Simons Center for Quantitative Biology, Cold Spring Harbor Laboratory, One Bungtown Road, Cold Spring Harbor, NY 11724, USA; 6 Eccles Institute of Human Genetics, University of Utah, 15 North 2030 East, Salt Lake City, UT 84112, USA; 7 Plant Genome Mapping Laboratory, University of Georgia, 111 Riverbend Road, Athens, GA 30602, USA; 8 Department of Crop and Soil Sciences, University of Georgia, 120 Carlton Street, Athens, GA 30602, USA; 9 J Craig Venter Institute, 9704 Medical Center Drive, 20850 Rockville, MD, USA; 10 School of Plant Sciences, iPlant Collaborative Bio5 Institute, University of Arizona, 1657 East Helen Street, Tucson, AZ 85745, USA; 11 Department of Horticulture, Michigan State University, A288 Plant and Soil Sciences Building, 1066 Bogue Street, East Lansing, MI 48824, USA; 12 Department of Microbiology, Immunology and Biochemistry, University of Tennessee Health Science Center, 858 Madison Avenue Suite G01, Memphis, TN 38163, USA; 13 Department of Chemistry and Biochemistry and Institute for Genomics and Proteomics, University of California, Los Angeles, 607 Charles E Young Drive East, CA 90095, USA; 14 Department of Biology and Intercollege Graduate Program in Plant Biology, The Pennsylvania State University, 201 Life Sciences Building, University Park, PA 16802, USA; 15 Center for Applied Chemical Biology, Department of Biological Sciences, Youngstown State University, 1 University Plaza, Youngstown, OH, 44555, USA; 16 USDA-ARS, Purdue University, 915 West State Street, West Lafayette, IN 47907, USA; 17 Texas A&M AgriLife Research, Department of Plant Pathology & Microbiology, Texas A&M University System, 17360 Coit Road, Dallas, TX 75252, USA; 18 Department of Biology, University of Central Oklahoma, 100 North University Drive, Edmond, OK 73034, USA; 19 School of Earth and Environmental Sciences, University of Adelaide, North Terrace, Adelaide, 5005, Australia; 20 Cryobiofrontier Research Center, Faculty of Agriculture, Iwate University, Ueda 3-18-8, Morioka, Iwate 020-8550, Japan; 21 Institute for Conservation Biology, The University of Wollongong, Northfields Avenue, Wollongong, NSW 2522, Australia; 22 Department of Crop Sciences, University of Illinois at Urbana-Champaign, 1101 West Peabody Drive, Urbana, IL 61801, USA; 23 Donald Danforth Plant Science Center, 975 North Warson Road, St Louis, MO 63132, USA; 24 Lawrence Berkeley National Laboratory, 1 Cyclotron Road Berkeley, Emeryville, CA 94720, USA; 25 Institute of Developmental Biology and Molecular Medicine & School of Life Sciences, Fudan University, 220 Handan Road, Shanghai, 200433, China; 26 Department of Biochemistry and Molecular Biology, 246 Noble Research Center, Oklahoma State University, Stillwater, OK 74078, USA; 27 Hawaii Agriculture Research Center, 94-340 Kunia Road, Waipahu, HI 96797, USA; 28 Department of Tropical Plant and Soil Sciences, University of Hawaii at Manoa, 3190 Maile Way, Honolulu, HI 96822, USA; 29 Fujian Normal University, Qishan Campus, Minhou, Fuzhou, 350117, China; 30 Department of Biology and Molecular Biology, Montclair State University, 1 Normal Avenue, Montclair, NJ 07043, USA; 31 Institute of Tropical Biosciences and Biotechnology, China Academy of Tropical Agricultural Sciences, 4 Xueyuan Road, Haikou, Hainan 571101, China; 32 Department of Plant and Microbial Biology, University of California, 1 Shields Avenue, Davis CA, 95161, USA; 33 Department of Cell and Developmental Biology, University of Illinois, 1201 West Gregory Drive, Urbana IL, 61801, USA; 34 The Genome Analysis Center, Monsanto, St Louis, MO 63167, USA; 35 Global Change and Photosynthesis Research Unit, Agricultural Research Service, United States Department of Agriculture, 1206 West Gregory Drive, Urbana, IL, USA; 36 IGPP Center for the Study of Evolution and Origin of Life, Geology Building, Room 5676, University of California, Los Angeles, 595 Charles E Young Drive East, Los Angeles, CA 90095-1567, USA; 37 Department of Plant and Microbial Biology, University of California, 411 Koshland Hall, Berkeley, CA 94720, USA

## Abstract

**Background:**

Sacred lotus is a basal eudicot with agricultural, medicinal, cultural and religious importance. It was domesticated in Asia about 7,000 years ago, and cultivated for its rhizomes and seeds as a food crop. It is particularly noted for its 1,300-year seed longevity and exceptional water repellency, known as the lotus effect. The latter property is due to the nanoscopic closely packed protuberances of its self-cleaning leaf surface, which have been adapted for the manufacture of a self-cleaning industrial paint, Lotusan.

**Results:**

The genome of the China Antique variety of the sacred lotus was sequenced with Illumina and 454 technologies, at respective depths of 101× and 5.2×. The final assembly has a contig N50 of 38.8 kbp and a scaffold N50 of 3.4 Mbp, and covers 86.5% of the estimated 929 Mbp total genome size. The genome notably lacks the paleo-triplication observed in other eudicots, but reveals a lineage-specific duplication. The genome has evidence of slow evolution, with a 30% slower nucleotide mutation rate than observed in grape. Comparisons of the available sequenced genomes suggest a minimum gene set for vascular plants of 4,223 genes. Strikingly, the sacred lotus has 16 COG2132 multi-copper oxidase family proteins with root-specific expression; these are involved in root meristem phosphate starvation, reflecting adaptation to limited nutrient availability in an aquatic environment.

**Conclusions:**

The slow nucleotide substitution rate makes the sacred lotus a better resource than the current standard, grape, for reconstructing the pan-eudicot genome, and should therefore accelerate comparative analysis between eudicots and monocots.

## Background

Sacred lotus, so named because of its religious significance in both Buddhism and Hinduism, belongs to the small plant family Nelumbonaceae, with only one genus, *Nelumbo*, and two species: *N. nucifera *(Asia, Australia, Russia) and *N. lutea *(eastern and southern North America) [1]. Lotus is in the eudicot order Proteales, which lies outside of the core eudicots (Figure S1 in Additional file 1); its closest relatives are shrubs or trees belonging to the families Proteaceae and Platanaceae. Lotus was a land plant that has adapted to aquatic environments.

Used as a food for over 7,000 years in Asia, lotus is cultivated for its edible rhizomes, seeds and leaves. Its buds, flowers, anthers, stamens, fruits, leaves, stalks, rhizomes and roots have been used as herbal medicines for treatment of cancer, depression, diarrhea, heart problems, hypertension and insomnia [2,3]. Its seeds have exceptional longevity, remaining viable for as long as 1,300 years, and its vegetative rhizomes remain healthy for more than 50 years [1,2]. The nanoscopic closely packed protuberances of its self-cleaning leaf surface have been adapted in Europe for the manufacture of a 'self-cleaning' industrial paint, Lotusan. The use of this paint results in the so-called lotus effect that is now widely advertised for self-cleaning automobiles, buildings and fabrics.

Here, we report the sequencing and analysis of the sacred lotus genome, which descends from the most ancient lineage of angiosperms. We have studied the evolutionary history of the genome and genes involved in relevant processes governing the unique features of this ancient land plant, including its adaptation to aquatic environments.

## Results

### Genome sequencing and assembly

We sequenced the genome of the sacred lotus variety 'China Antique' with 94.2 Gb (101×) Illumina and 4.8 Gb (5.2×) 454 sequences. The final assembly includes 804 Mb, 86.5% of the estimated 929 Mb lotus genome [4]. The contig N50 is 38.8 kbp and the scaffold N50 is 3.4 Mbp (Table S1 in Additional file 1). The largest 429 scaffolds account for 94.8% of the assembled genome and 98.0% of the annotated genes. Among the 39 plant genomes published to date, the median N50 scaffold length is about 1.3 Mb, making lotus the eighth best assembled genome (Table S2 in Additional file 1). We constructed a high-density genetic map using 3,895 sequence-based restriction-associated DNA sequencing markers and 156 simple sequence repeat markers [5]. The former were sorted into 562 co-segregating bins and a total of 698 informative markers were mapped into nine linkage groups for the eight lotus chromosomes, with one gap remaining between two linkage groups (Table S3 in Additional file 1). The nine anchored megascaffolds have a combined size of 543.4 Mb, accounting for 67.6% of the genome assembly, and they are mostly proportional to the karyotype of the lotus chromosomes (Figure S2 and S3 in Additional file 1). The high quality of the lotus genome assembly is largely due to the unexpected homozygosity of the 'China Antique' variety. Although lotus is an out-crossing plant, its cultivation and vegetative propagation via rhizomes over the past 7,000 years may have imposed a narrow genetic bottleneck. This could be partly the consequence of its unique feature, seed longevity, which might have further reduced the number of generations in its evolutionary history in addition to vegetative propagation. The estimated heterozygosity in 'China Antique' is 0.03%, lower than the 0.06% of the sequenced papaya cultivar 'SunUp' after 25 generations of inbreeding [6]. The estimated heterozygosity in the American lotus *N. lutea *'AL1' variety is 0.37%, also low.

### Repeat content of the sacred lotus genome

Repetitive sequences account for 57% of the assembled genome, including 47.7% recognizable transposable elements (Table S4 in Additional file 1). Unlike most plants, which exhibit relatively inconsequential non-long terminal repeat retrotransposons (approximately 1% of the genome) [7-9], such non-long terminal repeat retrotransposons contribute 6.4% to the lotus genome. Differing from other plants that usually have more Gypsy-like elements [9,10], Copia and Gypsy-like elements are comparable in copy number and genomic fraction in lotus. Most major DNA transposon families are detected in sacred lotus (occupying 16% of the lotus genome), albeit with more than 10-fold variation in relative abundance. An exception, the *Tc1/Mariner *super-family, is absent from both the lotus and grape genomes [7], suggesting the frequent loss of this family of elements. Surprisingly, *hAT *(*Ac/Ds*-like) elements contribute to nearly 7% of the lotus genome, represented by more than 100,000 copies, more than in any other sequenced plant genome. Of these, CACTA elements are least abundant (0.4%) while *MULE*, *PIF *and *Helitron *elements have amplified to a moderate degree (2.5%, 2.7% and 3.6%, respectively). The lotus genome further includes 1,447 Pack-mutator-like elements that carry genes or gene fragments [11]. Analysis using expressed sequence tags (ESTs) indicated that at least 10 Pack-mutator-like elements are expressed, suggesting that they may play functional roles.

### Genome annotation and gene expression

Following repeat-masking and annotation, we inferred 26,685 protein-coding genes in lotus, including all 458 core eukaryotic proteins [12]; 82% of the genes have similarity to proteins in SwissProt as identified by Basic Local Alignment Search Tool (E <0.0001). The average gene length is 6,561 bp with median exon and intron lengths of 153 bp and 283 bp, respectively (Table S1 in Additional file 1). The average gene density is one gene per 30 kb, with genes spread more evenly over the assembled genome than in many other plant genomes (Figure S2 in Additional file 1), which are characterized by gene-rich regions often found at the distal regions of chromosomes arms. A total of 12,344 ESTs were aligned to 11,741 gene models, and 174 alternative splicing events were identified from 164 genes involving 380 EST contigs (Table S5 in Additional file 1). Of the annotated genes in lotus, 22,803 (85.5%) show expression in rhizomes, roots, leaves or petioles based on RNAseq data (Figure S4 in Additional file 1). Expression of the remaining genes is likely confined to seeds, flowers and other unsurveyed tissues. Expression of 3,094 protein-coding genes was tissue-specific, including 1,910 genes showing expression only in rhizomes and 841 only in roots; 14,477 genes are expressed across all tissues surveyed. Of the 1,910 rhizome-specific genes, we found several AP2-like ethylene-responsive transcription factors, BTB/POZ domain-containing proteins, heat shock proteins, homeobox transcription factors, kinesins and pentatricopeptide repeat-containing proteins (PPRs) (Table S6 in Additional file 1). In lotus, 544 genes were annotated as PPRs, with 201 of these expressed in the four tissues tested, and 199 only expressed in the rhizome. PPRs have been identified as a group of RNA-binding proteins involved in RNA processing, stability, editing, maturation and translation in plants. Although the molecular mechanism of their function has not yet been elucidated, their broad expression in lotus rhizome is notable.

### Ortholog classification and ancestral gene content in eudicots

The protein-coding gene sets from lotus and 16 other sequenced angiosperm species were used to identify putative orthologous gene clusters with Proteinortho v4.20 [13]. A total of 529,816 non-redundant genes were classified into 39,649 orthologous gene clusters (orthogroups) containing at least two genes (Table S7 in Additional file 1). Of the 26,685 protein-coding genes in lotus, 21,427 (80.3%) were classified into 10,360 orthogroups, of which 317 contained only lotus genes.

From this gene classification, we estimate a minimum gene set of 7,165 genes in 4,585 orthogroups for eudicots (Table S7 in Additional file 1). The minimum gene set for core eudicots (7,559 genes in 4,798 orthogroups) is only slightly larger than the eudicot-wide set, suggesting that the minimal gene set of the eudicot-monocot ancestor (6,423 genes in 4,095 orthogroups) would add at least 490 orthogroups associated with the eudicots as a whole.

**Figure 1:**
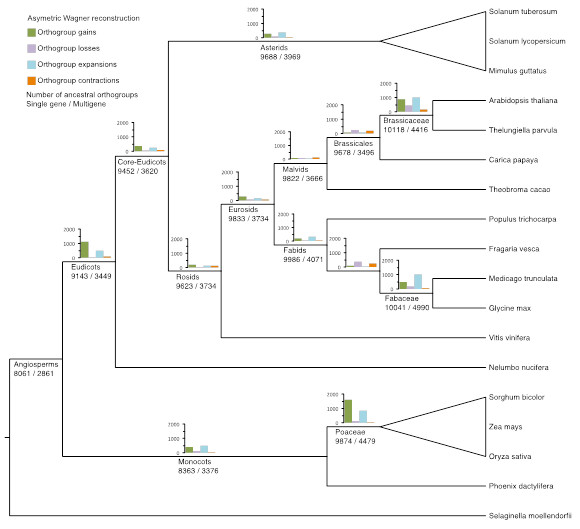
**Orthogroup dynamics in lotus and other angiosperm genomes**. Ancestral gene content and gene family (orthogroup) dynamics in lotus and other eudicot and monocot genomes identify expansion of the number of gene families and gene content associated with the ancestral eudicot.

We reconstructed the ancestral gene content at key nodes of the evolutionary series, as well as the adaptational changes occurring along the branches leading to these nodes: the greatest changes observed in orthogroup presence and absence are specific to terminal lineages (Tables S8 and S9 in Additional file 1 and Figure 1). More than three times as many orthogroup gains occur in the lineage leading to all eudicots, as compared to core eudicots (Figure S5 in Additional file 1), an increase second only to that of the grasses.

### Synteny and genome evolution

**Figure 2:**
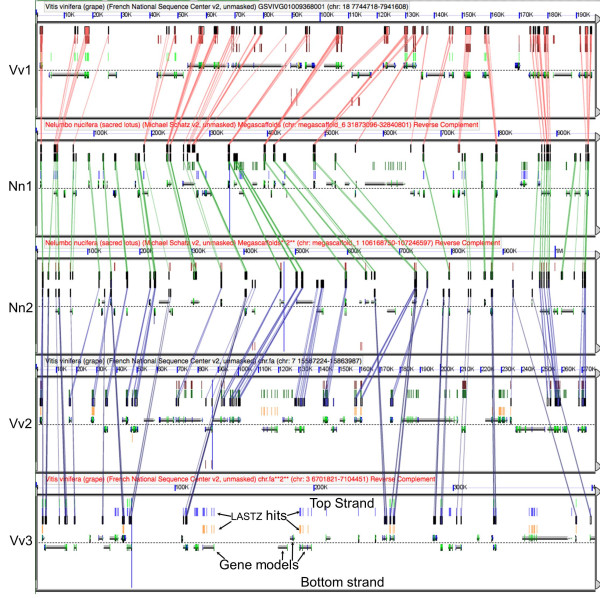
**High resolution analysis of syntenic regions of *Nelumbo nucifera *(Nn1/Nm2) and *Vitis vinifera *(Vv1/Vv2/Vv3)**. Synteny regions were identified from Figure S5 in Additional file 1. Gene models are arrays in middle of each panel; Colored boxes and lines connect regions of sequence similarity (LastZ) for protein-coding sequences between pair-wise comparisons.

A major evolutionary force shaping genome architecture in angiosperms is whole genome duplication (WGD) [14,15]. This process is followed by the 'diploidization' of genome organization through rearrangement, and of gene content through 'fractionation,' or homeologous gene loss. Intragenomic analysis of lotus indicates that it has experienced at least one WGD (paleotetraploidy, see Figure S6 in Additional file 1), named λ, but implies that the *Nelumbo *lineage did not experience γ, the paleohexaploidy (triplication) event around 125 million years ago detected in all other sequenced eudicot genomes [6,16-20]. Using lotus as a reference, as many as three post-γ grape subgenomic copies are equally evident, the syntenic regions of which show extensive collinearity of homologous genes (Figure 2). Among the 87.1% of the lotus genic regions retained from this duplication, 5,279 (33.3%) are singletons, 8,578 (54.1%) are duplicated, and 2,007 (12.6%) have more than three homeologs, implying there may have been additional paleo-duplications (Table S10 in Additional file 1).

Based on three lines of evidence, the lineage nucleotide substitution rate in lotus is about 30% slower than that of grape, widely used in angiosperm comparative genomics due to its basal phylogenetic position in rosids, slow mutation rate, and lack of reduplication. First, while phylogenetic evidence firmly dates the lotus-grape divergence before the pan-eudicot γ triplication affecting only grape, synonymous substitution rates (Ks) between genome-wide lotus-grape syntelog pairs (Figure S7 in Additional file 1) are smaller than those among triplicated grape genes. Second, the lotus lineage mutation rate also appears slower (about 29.26% slower) than that of *Vitis *based on a maximum-likelihood tree of 83 plastid genes [21] and expert dating of the respective speciation events [22] using the r8s program [23] with penalized likelihood. Third, the lotus genome has retained more ancestral loci following its lineage-specific WGD. Lotus is a basal eudicot, and its genome is the one from the most ancient lineage of angiosperm sequenced to date (Figure S1 in Additional file 1). Lotus represents an even better model than grape for inferences about the common ancestor of eudicots.

The remarkably slow mutation rate in lotus complicates the dating of the λ duplication. λ-duplicated lotus genes have a median synonymous substitution rate (Ks) of 0.5428, corresponding to an age of 27 million years ago (MYA) on the basis of average rates in plants [24] or 54 MYA on the basis of the grape lineage rate (Figure S7 in Additional file 1). Because lotus diverged from its closest sister lineage approximately 135 to 125 MYA [21], before the γ triplication, this suggests that the mutation rate in lotus is much lower than that in grape, and that the lotus-specific WGD event occurred about 65 MYA with a range between 76 and 54 MYA. This date coincides with the Cretaceous-Tertiary mass extinction that led to the loss of roughly 60% of plant species [25]. Polyploidization has been associated with increased adaptation and survivability, and the numerous plant species inferred to have undergone polyploidy within this time-frame suggests a possible advantage to polyploid lineages during the Cretaceous-Paleogene transition, an interpretation supported by the λ duplication in lotus.

**Figure 3:**
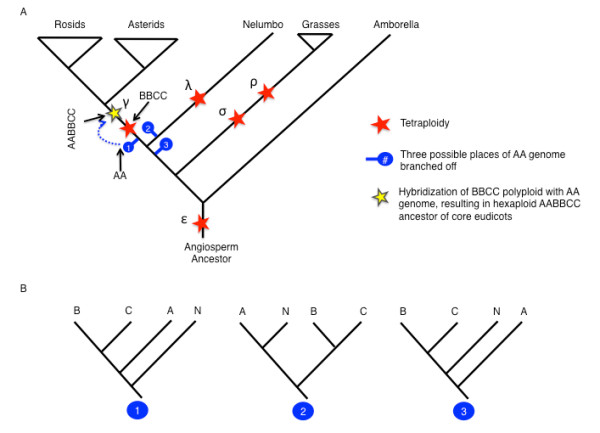
**Polyploidy events in the history of angiosperm evolution. (A)** Summary of polyploidy events in the history of angiosperm evolution, with a focus on the possible phylogenetic origins of the three subgenomes comprising the gamma paleohexaploidy event in core eudicots. Synteny analysis of the *Nelumbo *genome indicates that gamma is shared only within the core eudicots; however, phylogenomic analysis suggests a more complex history since around half of the gamma pairs were duplicated core-eudicot-wide and the other half eudicot-wide (See Table S10 in Additional file 1). AA, BB, and CC are three subgenomes of the ancestral hexaploidy. Three possible phylogenetic origins of the ancestral AA genome involved in gamma are denoted by 1, 2 and 3. Lamda is defined as the most recent polyploidy event in the evolutionary history of *Nelumbo*. All the other Greek symbols are well-known polyploidy events in the evolutionary history of angiosperms. Gamma: genome-triplication (hexaploid) event in core eudicot genomes [7,23]; Sigma and rho: genome duplications detected in grass genomes [8]; Epsilon: angiosperm-wide duplication detected in large-scale gene family phylogenies. Based on gene tree phylogenomics, we hypothesize that the triplication event involved a tetraploid event (BBCC red star) first, then subgenome AA combined with BBCC to form hexaploidy AABBCC (blue dashed line). **(B) **Predicted gene tree topologies of hypothetical origins of the AA subgenome of the gamma paleohexaploidy. A, B, C indicate surviving genes inherited from AA, BB, CC subgenomes of the AABBCC ancestral hexaploidy. N indicates genes of *Nelumbo*.

By tracing the phylogenetic histories of 688 pairs of grape genes in 528 orthogroups from each of the γ duplication blocks [26], we tested the timing of the γ paleohexaploid event that has been observed in the genomes of *Vitis *[7], papaya [6], *Populus *[20] and other core eudicots [14,17]. About 50% of the resolved trees support the timing of the γ event to have occurred 'core-eudicot-wide' after the divergence of lotus, consistent with synteny analysis. By contrast, gene family phylogenies for about half of the γ block duplications include lotus genes (Table S11 in Additional file 1), although, in rare cases, duplicated monophyletic groups contain both lotus and eudicot-wide genes. This is consistent with an earlier phylogenomic analysis using data from numerous plant genomes and basal eudicot transcriptomes, suggesting that 18% to 28% of γ block duplications were eudicot-wide [26], even though the signal is primarily observed in core eudicots (Figure 3).

Such data suggest that a relatively large amount of genetic novelty is specifically associated with eudicots as a whole, even though the core eudicots shared a genome-triplication after divergence from the basal eudicots. By contrast, in monocots it appears that the evolution of the grass family specifically, rather than the earlier node comprised of grasses (Poales) and palms (Arecales), was associated with relatively large gains in gene family number and size.

### Adaptation to an aquatic environment

**Figure 4:**
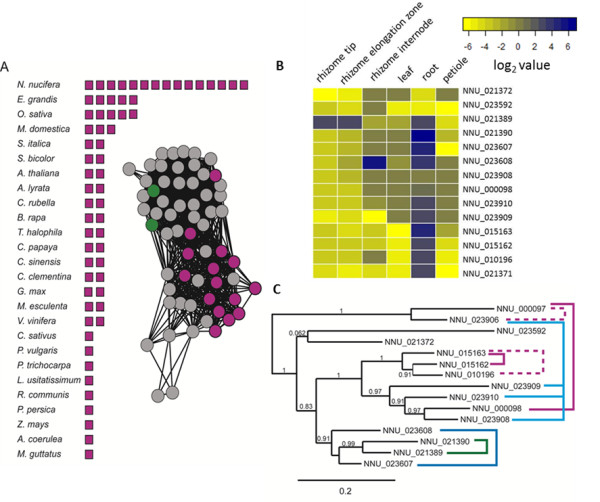
**Lotus-specific expansion in LPR1/LPR2 proteins**. (A) The number of LPR1/LPR2 homologs in land plants. Homologs detected by Basic Local Alignment Search Tool against the genomes of land plants are represented by a box. A protein similarity network of those proteins is also shown; lotus proteins are represented as purple nodes, Arabidopsis proteins (LPR1 and LPR2) are represented as green nodes and other land plant proteins are represented as grey nodes. **(B) **Heatmap of COG2132 gene family member expression in lotus. Reads per kilo base per million (RPKM) values were log_2 _transformed, where blue correlates to high expression, and yellow to low expression. **(C) **A maximum-likelihood tree of LPR1/LPR2-like lotus proteins. Branch support was calculated using an Approximate Likelihood-Ratio Test. Lotus homologs are connected with a dashed bracket, whereas proteins whose genes are found in tandem on the genome are connected with a solid bracket. A detailed phylogeny of COG2132 members can be found in Figure S8 in Additional file 1.

Submersed plant growth presents unique physiological challenges. Lotus has had to evolve novel features to cope with its aquatic lifestyle. Possible adaptations include an astonishing number of putative copper-dependent proteins, of which 63 proteins contain at least one COX2 domain, 55 contain a 'copper-binding-like' domain, and 4 contain polyphenol oxidases. The abundance of copper proteins in lotus compared to other plants is attributed to expansions in COG2132, a family of multi-copper oxidases. Most plant genomes encode one or two members of COG2132, whereas lotus has at least 16 members due to WGD and repeated tandem duplications (Figure 4, and see Figure S8 in Additional file 1). The only COG2132 members in Arabidopsis, LPR1 and LPR2, are involved in phosphate starvation signaling in root meristems. Similarly, in lotus, expression of COG2132 family members is confined largely to the roots (Figure 4). The lotus-specific expansion appears to form a separate phylogenetic clade from the LPR1 and 2-like proteins, suggesting a novel function not found in Arabidopsis (Figure 4, and see Figure S8 in Additional file 1).

Adaptation to phosphate starvation in lotus is also evidenced by expansion of the UBC24 family and the miR399 family that regulates it (Table S12 in Additional file 1). The miR169 family, implicated in adaptation to drought stress in Arabidopsis [27], also shows expansion in lotus, totaling 22 members. The fact that lotus grows aquatically and may rarely be subjected to drought suggests that the miR169 family is involved in other physiological processes.

Several other gene families also show unusual compositions that may reflect adaptation to aquatic lifestyles. The basic helix loop helix (bHLH) family, implicated in light responses including germination, control of flowering and de-etiolation, and root and flower development, lacks three of its 20 subfamilies in lotus: Va, implicated in brassinosteroid signaling; VIIIc2, implicated in root hair development; and XIII, implicated in root meristem development [28]. The largest families of bHLH factors in lotus are XII, involved in developmental processes including control of petal size, brassinosteroid signaling and floral initiation, and Ia, implicated in stomatal development and patterning.

The PRR1/TOC1 circadian clock family, which coordinates internal biology with daily light/dark cycles and is highly conserved across many plant species, includes three predicted members in lotus compared to the one or two present in other plant genomes. The fact that PRR proteins have key roles in modulating light and temperature input into the circadian clock suggests that lotus may require more sensitive adjustments to its environment than other plants. Consistent with this, the cryptochrome (CRY) family of blue light photoreceptors is also increased with five (two CRY1, two CRY2, one CRY3) compared to three in Arabidopsis and four in poplar (Additional file 1, Table S13). Similar expansion in the CRY family was also noted in another aquatic organism, *Ostreococcus*, a micro green algae. Lotus is adapted to both temperate and tropical climates and day lengths with a wide range of flowering times, perhaps associated with increased numbers of flowering time and circadian clock-associated genes.

## Discussion

Paleopolyploids are widespread among eukaryotes and particularly common in angiosperms [14,15]. Lotus diverged from other eudicots early in eudicot history, prior to the γ genome-triplication characteristic of most members of the group [14,15,17,26], and provides insight into the timing and nature of this event associated with a rapid radiation of the large eudicot lineages. When plant genomes of high paleopolyploidy levels are compared, differentiated gene loss (fractionation) among several homologous subgenomes tends to diminish the signals of synteny. In such cases, genomes with few paleopolyploidy events (such as those of grape or papaya) can be used to take advantage of the smaller evolutionary distances between orthologous segments. Extensive collinearity within itself, as well as with other plant genomes such as those of Arabidopsis, grape, rice and sorghum, makes the lotus genome not only a eudicot evo-genomic reference (Figure S9 in Additional file 1), but also a better resource for reconstructing the pan-eudicot genome and facilitating comparative analysis between eudicots and monocots.

Surprisingly, the phylogenomic analysis of gene families associated with the γ include a substantial fraction of eudicot-wide duplications, suggesting the possibility of a two-step model that involved genetic material from a lineage that branched off earlier than the core eudicots (Figure 3A). A substantial fraction of eudicot-wide gene duplications was also observed in phylogenomic analyses that contained large collections of transcriptome data from early branching basal eudicots such as *Platanus*, *Aquilegia *and poppies [26]. Eudicot-wide duplications were detected only rarely in another phylogenomic analysis that introduced transcriptome data from the basal eudicots *Gunnera *and *Pachysandra *[29]. The 34 unigenes available from that study were used to populate five MADS box orthogroups with larger taxon sampling in this study. Phylogenies of these orthogroups identify (at boostrap >50%) one eudicot-wide and three core-eudicot-wide duplications (Table S11 in Additional file 1), consistent with the rest of the findings in the present study.

In contrast to the phylogenomic results, syntenic comparison showed one lotus region matched with up to three *Vitis *homologous regions, indicating that the lotus genome did not share the γ event. We propose that the γ event occurred after the separation of the lotus lineage (Proteales), and involved hybridization with a now extinct species that branched off around the same time (Figure 3A, AA at position #2), or even earlier than lotus (Figure 3A, AA at position #3). This model explains why the phylogenomic analyses could identify some γ duplications occurring before the divergence of lotus, but not observable as a triplication in the lotus genome structure. A similar two-step model was suggested by Lyons *et al. *[30] on the basis of fractionation patterns seen in *Vitis*, and evidence for a two-step hexaploid process is clearly observed in the much more recent paleohexaploid *Brassica rapa *[31]. Additional whole plant genome sequences from lineages close to the γ event, especially ones without the confounding effects of lineage-specific genome duplications, may also help to clarify genome-wide patterns of fractionation among the three γ subgenomes, which could provide further evidence bearing on the timing and event(s) associated with the γ paleohexaploidy event that is associated with what is arguably one of the most important radiations in angiosperm history.

The higher homeolog retention rate in lotus compared with most other genomes studied provided an opportunity to study subfunctionalization [32], a major driving force affecting fates of duplicated genes following paleopolyploidy. Most pairs of lotus homeologs have no difference in PFAM domain families, whereas 453 pairs (11.6%) differ by up to five domains. The unshared domains have mean length 17 amino acids with a range of 0 to 890 amino acids. Between homeologous lotus gene pairs, mRNA length (excluding 5′ and 3′ untranslated regions), coding sequence length, and intron length differences all follow geometric-like distributions (Figure S10 in Additional file 1), consistent with independent accumulation of small insertions and deletions. The changes of length in exonic and intronic regions seem uncorrelated, implying that subfunctionalization affects gene regulation at multiple transcriptional and post-transcriptional levels.

When divergence of lineages is followed by WGD, one predicts similar divergence of the paralogs in one species' genome from a shared ortholog in the other species, confirmed in previous studies [16,33]. Comparison of paired λ paralogs and their grape ortholog generally fit this prediction (Figure S11 in Additional file 1); however, comparisons to cereal (sorghum) orthologs show consistent differentiation in branch lengths. This discrepancy in the lotus-cereal comparison could be explained by fast evolutionary rates in cereal genomes and/or λ being older than it appears, due to the slow *Nelumbo *evolutionary rate. Alternatively, this is also consistent with structural compartmentalization, with genes within the same genome undergoing different evolutionary trajectories [33]. Wider taxa sampling at neighboring branches will help better distinguish the possibilities.

The extraordinary seed longevity and vegetative propagation via rhizomes are likely the causes of the slow evolutionary rate in lotus. The 'China Antique' has a highly homozygous genome, yielding arguably the best assembled genome using next-generation sequencing technologies with pseudo-molecules proportional to its karyotype. The lotus genome provides the foundation for revealing the molecular basis of its many distinguishing biological properties, including seed longevity, adaptation to aquatic environment, the distinctive superhydrophobicity and self-cleaning property of its leaves, and the thermogenesis that is thought to enhance its pollination success.

Sacred lotus is the first true aquatic plant to be sequenced and comparative genomics reveal unique gene family expansions that may have contributed to its adaptations to an aquatic environment. Submersed soils are largely hypoxic and have a decreased reduction-oxidation potential, causing heavy metal precipitation and reduced nutrient availability. Lotus has a dramatic expansion of the COG2132 family, a group of multi-copper oxidases involved in phosphate starvation in root meristems. A role in root-specific processes is supported by the expression of these unique genes in root tissue. Adaptation to phosphate starvation can also be seen in an expansion of the UBC24 family and the miR399 family that regulates it. Lotus lacks four bHLH subfamilies involved in iron uptake and root hair and root meristem development, suggesting novel root growth and iron regulation. These gene family expansions and preferential retention of duplicated genes reflect the challenges of aquatic growth.

## Conclusions

Sacred lotus has many unique biological features, most noticeable seed longevity and the lotus effect, in addition to its agricultural and medicinal importance. The purpose of sequencing the lotus genome is to facilitate research in these areas and on agronomic and horticultural traits such as rhizome development and flowering time. The assembly of the lotus genome is surprisingly high quality, largely due to the high level of homozygosity resulting from domestication and vegetative propagation. The lotus genome has a lineage-specific WGD event that occurred about 65 MYA, but shows no structural evidence for the γ hexaploid event shared among core eudicot species. The lotus genome has a 30% slower nucleotide mutation rate than that of grape, contributing in part to the outstanding genome assembly using next-generation sequencing technologies. Analysis of sequenced plant genomes yielded a minimum gene set for vascular plants of 4,223 genes. Strikingly, lotus has 16 COG2132 multi-copper oxidase family proteins with root-specific expression. COG2132 members are involved in root meristem phosphate starvation, reflecting lotus' adaptation to limited nutrient availability in an aquatic environment. The slow nucleotide substitution rate and the lack of the triplication event make lotus genome an excellent reference for reconstructing the pan-eudicot genome and for accelerating comparative analysis between eudicots and monocots. The lotus genome will accelerate the identification of genes controlling rhizome yield and quality, seed size and nutritional profile, flower morphology, and flowering time for crop improvement.

## Materials and methods

Illumina (Illumina HiSeq 2000) libraries were generated from purified *N. nucifera *'China Antique' nuclear DNA with inserts of 180 bp, 500 bp, 3.8 kb and 8 kb and assembled using ALLPATHS-LG. 454/Roche (GSFLX pyrosequencing platform) 20 kb mate pair reads were used for scaffolding. RNAseq data generated from various lotus tissues were used for annotation and RNAseq differential gene expression analysis using CLC Genomics Workbench 5.0 (CLC Bio, Aarhus, Denmark). MAKER version 2.22 was used in combination with the assembled RNAseq data to annotate 26,685 genes in the lotus genome. Detailed methods for genome assembly, annotation and analyses are provided in Additional file 1.

### Data access

The assembled *N. nucifera *genome was submitted to GenBank (AQOG00000000; PID PRJNA168000, http://www.ncbi.nlm.nih.gov/Traces/wgs/?val=AQOG01). Whole genome shotgun raw reads are deposited under SRA study: SRP021228 (http://trace.ncbi.nlm.nih.gov/Traces/sra/?study=SRP021228). The raw RNAseq data are deposited under BioProject 196884 (http://www.ncbi.nlm.nih.gov/bioproject/196884).

## Abbreviations

bHLH: basic helix loop helix; bp: base pair; CRY: cryptochrome; EST: expressed sequence tags; MYA: million years ago; PPR: pentatricopeptide repeat-containing proteins; WGD: whole genome duplication.

## Authors' contributions

RM, RV, YL, MY, YH and S L designed research; RM, RV, YL, MY, YH, LTL, QZ, JEB, HT, EL, AAF, GN, DRN, CEBH, ARG, YJ, JPD, FZ, JH, XM, KAH, KI, SAR, MEH, QY, TCM, AC, YZ, RS, RJ, NC, JA, CMW, EW, AS, YH, LX, JZ, RP, MJH, WX, JAW, JW, MLW, YJZ, REP, ABB, CD, SRD, MAS, TPM, SPL, DRO, JWS, DRG, NJ, MY, CWD, SSM, AHP, BBB, SL and JSM performed research and analyzed data; RM, RV, JL, AHP, CEBH, JRW, KI, SAR, CWD, SSM and BBB wrote the paper. All authors read and approved the final manuscript.

## Supplementary Material

Additional file 1 **Supplementary data, including detailed materials and methods, and supplementary tables S1-S13, and figures S1-S14**. Click here for file
