# Supplementary material for: Genome of the long-living sacred lotus (Nelumbo nucifera Gaertn.)
Source: Genome Biol. 2013 May 10;14(5):R41. doi: 10.1186/gb-2013-14-5-r41 (PMC4053705; doi:10.1186/gb-2013-14-5-r41)
Supplement: Additional file 1 — Supplementary data, including detailed materials and methods, and supplementary tables S1-S13, and figures S1-S14. [file gb-2013-14-5-r41-S1.DOC]

**SI appendix**

**Table of contents:**

**Materials and Methods**

**Nuclear DNA preparation**

**Genetic map construction**

**Estimation of residual heterozygosity**

**Genome sequencing and assembly**

**Transcriptome assembly**

**Gene prediction and annotation**

**RNAseq expression analysis**

**Construction of lotus repeat database**

**Synteny analysis**

**Global gene family classification and phylogenetic analysis**

**Comparison of lineage nucleotide substitution rates in *Nelumbo* and *Vitis***

**Alternative splicing**

**Financial Support**

**Supplemental tables S1-S13**

**Supplemental figures S1-S13**

**Methods**

**Nuclear DNA preparation**

Fresh leaf tissues were collected the leaves in the extraction buffer for 10 min, the suspension was filtered through two layers from etiolated lotus'China Antique' seedlings cultured under dark conditions. Tissues were frozen in liquid nitrogen and stored at -80°C before use. Nuclei were prepared using the procedure outlined in [34] with modifications. The HB (1X) nuclei extraction buffer (10 mM Tris base, 80 mM KCl, 10 mM EDTA, 1 mM spermidine, 1 mM spermine, 0.5 M sucrose, 0.15% β-mercaptoethanol, pH 9.4-9.5) was modified by adding PVP-40 at a ratio of 2% (w/v). After incubating of cheesecloth and one layer of miracloth and centrifuged at 1800 g for 20 min. The nuclei were then washed twice with wash buffer (1X HB plus 2% Triton X-100), each wash step consisting of centrifugation at 1800 g for 20 min.

After the final wash, the pelleted nuclei were resuspended in the DNA extraction buffer (50 mM Tris base, 5 mM EDTA, 710 mM NaCl, 350 mM sorbitol, 1% SDA, 0.1% CTAB, 0.5 M sucrose, 0.10% β-mercaptoethanol, pH 8.0 ). After incubation at 65°C for 30 min, the slurry was centrifuged at 12000 rpm for 8 min and the supernatant was extracted with the same volume of chloroform : isoamylalcohol (24:1, v/v), then centrifuged at 12000 rpm for 8 min. The same volume of -20°C pre-cooled isopropanol was added to the supernatant, mixed and incubated at -20°C for 30 min. After centrifugation, the pellet was washed with 600 µl of 75% ethanol, air dried at room temperature and resuspended in 200 µl Tris EDTA (TE) buffer [10 mM Tris-HCl, 1 mM ethylenediaminetetraacetic acid (EDTA), pH 8.0] containing 10 ng/µl ribonuclease A.

**Genetic map construction**

An F1 population with 43 individuals was used to generate the integrated genetic map for anchoring scaffolds, which was derived from a cross between Chinese lotus ‘China Antique' (*Nelumbo nucifera*) and American lotus 'AL1' (*N. lutea*). Markers were generated from Restriction Associated DNA sequencing (RADseq). Parental and progeny genomic DNA was isolated from fresh leaf tissues. RADseq libraries were constructed using double restriction endonucleases, *Nsi* I (5’ATGCA/T3’) and *Mse* I (5’T/TAA3’). The digested DNA fragments were then ligated to adapter 1 and adapter 2 at the same time; adapter 1 contained the recognition site of *Nsi* I followed by a 4 to 6 nucleotides barcode, and adapter 2 contained the recognition site of *Mse* I, and a 6 nucleotide Illumina TruSeq index. After size selection, the adapter ligated DNA fragments were enriched by PCR amplification. The RAD Libraries were normalized to 10 nM and sequenced using an Illumina HiSeq2000 instrument following the standard protocols. On average, 153 Mbp of sequences were obtained per individual (6.9 Gbp of 92 bp reads total) and trimmed reads were used for Single Nucleotide Polymorphism (SNP)/Insertion Deletion (InDel) detection and scoring. The repeat masked scaffold sequences were used for read alignment to reduce erroneous marker detection caused by high copy number repetitive elements. SNP/InDel markers with the segregation type of homologous loci in 'China Antique' and heterozygous loci in 'AL1' were used in linkage mapping. SNP/InDel calling was performed using a custom protocol, which combined Stacks package, Novoalign, SAMtools, a custom Shell and Perl scripts with a minimum three reads of the same SNP or InDel to score them as segregating markers.

4098 RADseq markers and 136 SSR markers were used for genetic map construction. The 4098 RADseq markers were assigned to 634 recombination bins, of which 562 (3895 RAD markers) were integrated with 136 SSR markers to construct an American lotus genetic map. Mapping was conducted using the CP population model in JoinMap 4.1, with regression mapping algorithm and Kosambi’s mapping function. Markers were assigned to linkage groups with thresholds of a LOD score at 5.0 and a recombination rate at 0.4. The total distance of the genetic map is 494.3 cM on 9 linkage groups, with an average distance of 0.7 cM between adjacent markers. The longest linkage group is 97.7 cM, and the shortest is 21.5 cM. The high density genetic map anchored 71% of the assembled genome, missing regions that are largely monomorphic such as the 43 Mb megascaffold 6 with merely 8 mapped RADseq markers in three bins.

**Estimation of residual heterozygosity**

Heterozygosity of the lotus genome was estimated using RADseq data. The aligned length of all RAD reads against the lotus scaffolds were summed, and regions with greater than 3X coverage were assessed for SNPs using a custom Perl script. Each SNP was accepted based on the ratio of the two alternative nucleotides, with chi-square test at 1% level of significance. Heterozygosity was calculated by dividing the number of high confidence SNPs by the total length of aligned RAD reads.

**Genome sequencing and assembly**

Raw sequences were generated primarily using Illumina sequencing, following standard protocol with the HiSeq 2000. Four paired-end libraries were created with inserts of 180bp, 500bp, 3.8kb and 8kb, generating 33x, 35x, 6.4x, and 6.1x coverage, respectively. A paired-end 20kb insert library was generated for scaffolding using Roche/454 circularization protocol with sequencing carried out on the 454 FLX+.

Before assembling the data, we evaluated the lotus genome heterozygosity by examining the frequency of kmers (19-mers) in the unassembled lotus reads and of the F1 (fig. S12). This analysis is very sensitive for measuring heterozygosity because heterozygous sequences will be sampled at approximately half the depth as homozygous sequences. The kmer coverage distribution of the lotus genome showed a unimodal, approximately Gaussian distribution centered at ~70x coverage, as expected by the genome size and total sequence coverage from which we conclude the genome does not have a substantial rate of heterozygosity. In contrast, an F1 cross between lotus and *N. lutea*, which is expected to be heterozygous, was clearly bimodal with peaks at both 40x representing the homozygous regions and 20x representing the heterozygous kmers. In addition to the main peaks, both distributions also showed high coverage repeats, as expected for a genome with high repeat content.

An initial assembly of the data, excluding the 20kbp library, was created using ALLPATHS-LG [35] and resulted in an N50 contig and scaffold size of 25kbp and 600kbp, respectively. The assembly used the default ALLPATHS-LG parameters and routines for error correction, contiging, and scaffolding. We selected ALLPATHS-LG based on our prior positive experiences with it in the Assemblathon and the GAGE evaluations[36, 37]. The final assembly was a hybrid assembly using a combination of the Illumina and 454 sequencing reads, especially to use the 20kbp library to improve the scaffolding. However, since ALLPATHS-LG does not natively support 454 data files or have capabilities to correct 454 error types, we first converted the reads into an acceptable format using our routines developed as part of the AMOS assembly software package [38]. In particular, 454 pyrosequencing is known to have a high rate of homopolymer sequencing errors that are not supported by the ALLPATHS-LG error correction routines. As such we first used sff_extract to extract the mate pairs containing the expected linker sequence from the raw sff sequencing files. We then trimmed the 3’ ends of the reads to 40bp to minimize any homopolymer error effects, and aligned the reads to the draft Illumina-only assembly using BWA [39]. Finally, using the alignextend routine developed and distributed with AMOS, we computationally extended the 3’ ends of the reads using the consensus sequence of the draft assembly into 100bp reads and output them into FASTQ format as required by ALLPATHS-LG. This procedure is highly effective for correcting errors with the data: homopolymer errors and other sequencing errors are in essence “corrected” by replacing the read with the consensus of the Illumina-only assembly; PCR induced duplicate pairs are identified and discarded based on their alignment position to the draft assembly; and very low quality mate-pairs that fail to align to the assembly are discarded completely.

After these cleaning routines were complete, we reassembled the genome again using ALLPATHS-LG (MIN_CONTIG=300, but otherwise default parameters). Since by design no new sequence was introduced to the assembly, the contig N50 only marginally improved to 38.8kbp, but the scaffold N50 size jumped nearly 7 fold to 3.43 Mbp, including 10 scaffolds longer more than 10Mbp (max: 14.3Mbp). The assembled genome was submitted to GenBank under genome ID 14095: <http://www.ncbi.nlm.nih.gov/genome/14095>

As a final assembly improvement routine, we assembled the scaffolds into “megascaffolds”, based on the American and Chinese lotus genetic maps, synteny to the *Vitis vinifera* assembly, and any additional long range pairs that we could identify. This information was used to order and orient the sequence scaffolds into larger megascaffolds. Markers in each co-segregating bin anchoring to different scaffolds were used to add scaffolds to megascaffolds. Most scaffolds joined in the megascaffolds were based on multiple lines of evidence, allowing scaffolds to be ordered and oriented as described for the *Sorghum bicolor* [8].

**Transcriptome assembly**

RNAseq data was generated using both Illumina and 454 sequencing platforms. For constructing the Illumina RNAseq libraries, total RNA was first extracted from rhizomes using the RNeasy mini kit (Qiagen, Valencia, CA). The transcriptome libraries for 100bp paired-end (PE) sequencing were made with the TruSeq RNA Sample Prep Kit v1 (Illumina, San Diego, CA) according to manufacturer’s instructions. The library samples were clustered on a flow cell using the cBOT and the flow cell was loaded on the Illumina HiSeq2000 sequencer for sequencing at Macrogen (Macrogen, Seoul, Korea). Initial base calling and quality filtering of the Illumina sequencing image data were performed using the Illumina pipeline CASAVA v1.8.2. The raw sequencing reads were trimmed with quality value ≥ 30, and short reads less than 20 bp were removed for the subsequent analysis. The filtered reads were assembled using CLC Genomics Workbench 5.0 (CLC Bio, Aarhus, Denmark) with default settings then, potential poly-A tails were removed with EMBOSS trimest [40] followed by finalizing with MIRA [41] and CAP3 [42], which resulted in 207,965 contigs.

RNA for 454 transcriptome library construction was isolated using methods previously described by [43] and pooled with equimolar concentrations of RNA extracted from germinating seeds, open flowers, rhizomes, roots, petioles, floating and aerial leaves. 5 ug of synthesized cDNA was used for GS FLX library preparation following standard Roche protocol. After removing low quality reads, 680,000 reads were assembled using GS Denovo Assembler with a minimum overlap of 40bp and a stringent identity of 95%. A total of 16,349 nonredundant EST contigs were assembled from 454 EST reads.

**Gene prediction and annotation**

MAKER version 2.22 [44] was run on lotus using assembled mRNA-seq data, and all NP id containing RefSeq plant proteins as evidence (downloaded November 17, 2011 from ftp://ftp.ncbi.nih.gov/refseq/release/plant). Repetitive regions were masked using a custom repeat library, all organisms in Repbase [45], and a list of known transposable elements provided by Repeatmasker. Additional areas of low complexity were soft masked [46] using Repeatmasker to prevent the seeding of evidence alignments in those regions but still allowing extension of evidence alignments through them [47, 48]. Genes were predicted using SNAP [48] and Augustus [49, 50] trained for  *lotus* using MAKER in an iterative fashion as described by Cantarel et al. [47]

The final annotation set contains 26,685 protein coding genes with 71%containing a protein domain as detected by IPRscan [51], and 95% of which have an annotation edit distance less than 0.5, consistent with a well annotated [8]. In addition all 458 core eukaryotic proteins identified by Parra et al. (2007), are represented in the final annotation set and 82% of the annotated genes have similarity to proteins in SwissProt as identified by BLAST [52] (E < .0001). The average gene length is 6,561bp with median exon and intron lengths of 153bp and 283bp respectively.

**RNAseq expression analysis**

Trimmed sequence reads from the rhizome (tip, internode, and elongation zone), leaf, petiole, and root libraries were mapped to the transcripts, and the total number of reads mapping to each transcript was counted using CLC Genomics Workbench. A RPKM (Reads Per Kilobase of exon model per Million mapped reads) value was calculated for each transcript to determine the expression level in the tissues [53]. Genes were considered to be expressed in a tissue if they had RPKM values ≥1 in that tissue [54]. This led to the determination that 22,803 genes were expressed in at least one of four different tissues: 20,866 in rhizome, 16,656 in leaf, 19,457 in root and 16,845 in petiole (fig. S3). 14,477 genes were expressed in all tissues. The tissue with the largest number of expressed genes was the rhizome. In all, 3,094 genes were expressed in a tissue-specific manner: 1,910 in rhizome, 232 in leaf, 841 in root, and 111 in petiole.

**Construction of lotus repeat database**

Repetitive sequences in the genome of lotus were collected with a variety of approaches. LTR elements were collected through LTRharvest [55], with parameters “-minlenltr 80 -maxlenltr 6000 -mindistltr 300 -mintsd 4 -maxtsd 6 -motif tgca -similar 90”. The resulting elements were further screened using LTRdigest [56] for the presence of a poly purine tract (PPT) or primer binding site (PBS). Only elements with a PPT or PBS were retained. Subsequently, 100 bp of flanking sequences (5’ and 3’) of the LTRs of these elements were retrieved and aligned with dialign 2 [57]. If 50 bp or longer of the flanking sequences of a single element was aligned at a similarity of 60% or higher, the boundary of the element was considered to be incorrect, and the element was excluded. The remaining elements were considered to be bona fide LTR elements. To reduce the redundancy, examplar elements were selected using script “examplar_maker.pl” which is from the MITE-Hunter package [58]. Non-autonomous “cut and paste” DNA elements including MITEs were collected using MITE-Hunter with recommended parameters in the manual. Terminal inverted repeats (TIRs) of *Mutator*-like elements were identified as described previously [59]. The sequences of exemplars of LTR elements and those of non-autonomous DNA elements, and MULE TIRs were used to mask genomic DNA, and repetitive sequences in the unmasked portion of the genomic DNA were identified using RepeatModeler (<http://www.repeatmasker.org/RepeatModeler.html>).

The output of RepeatModeler contains both repeats with identity (known repeats) and unknown repeats. After filtering putative gene families (sequences matching non-transposase proteins, E< 10^-5^), repetitive sequences with a copy number higher than 1000 were manually curated to verify their identity and 5’/3’ boundaries, as follows: first, the relevant sequence was used to search the lotus genomic sequences and at least 10 hits (BLASTN, E< 10^-10^) with the corresponding 100 bp of 3’ and 5’ flanking sequences were recovered. Recovered sequences were then aligned using dialign 2 [57], with the resulting output examined for the presence of possible boundary between putative elements and their flanking sequences. A boundary was defined as the position to which sequence homology is conserved over more than half of the aligned sequences (*e.g.*, 6 of 10 sequences), and sequences at the boundary of the putative element were compared with that of a known transposable element (TE). Furthermore, the sequences immediately flanking the element boundaries were examined for the presence of target site duplication, which is created by most transposons upon insertion. Each transposon family has unique terminal sequences and target site duplication, which can aid in the identification of a specific transposon [60]. For some large transposable elements, fragmented sequences identified by RepeatModeler were joined to derive a compete sequence. If a particular sequence is similar to a known transposon at the nucleotide level or protein level (BLASTX or BLASTN E< 10^-5^, RepBase17.02), it is considered to be the relevant TE. Finally, the putative terminal sequence was aligned (directly and inversely) using “gap” in GCG package to detect possible inverted or direct repeats. All the above information was used to determine the identity of each specific repeat.

Manually curated sequences were compared to the unknown repeats using RepeatMasker. Sequences matching the curated sequences were considered to belong to the same repeat family and excluded. The criteria for exclusion were as follows: if two elements share 80% or higher similarity in 90% of their element length, they are considered to be the same family. If a repetitive sequence matches the curated sequences without reaching the above criteria, this sequence is retained and is considered to belong to a new family within the same superfamily. All sequences of curated and non-curated repeats were used as a repeat library to mask the genomic sequence for their coverage and copy number. If an element in the genomic sequence matched a sequence in the repeat library over the entire sequence, or if the truncation was less than 20 bp on each end, this copy was considered to be intact. Otherwise it was considered as a truncated sequence or half of a copy. Fragmented elements that lack both ends (truncated more than 20 bp on both ends) were not included in copy number estimation. The genome coverage of TEs was estimated as the total sequence masked by each superfamily with overlapping regions only calculated once.

Pack-MULE elements (*Mutator*-like elements carrying genes) were identified as described [61]. The element sequences were compared with EST database to search for evidence of expression. If a Pack-MULE element matches an EST sequence with 97% or higher similarity and the Pack-MULE coordinate is the best hit for the EST in the genome, these elements are considered to be expressed.

*Helitrons* were predicted by an improved version of HelitronFinder [62, 63] which is a work in progress. The new version is developed based on the Local Combinational Variable (LCV) algorithm [64]. It first draws LCVs from 5’ and 3’ ends of known Helitrons, and then scans the whole genome while scoring with LCV hits. Putative *Helitrons* are from regions with scores above predefined threshold at both ends.

**Synteny analysis**

Alignment between the lotus and grape genomes was performed by LAST [65] with default settings. Gene homology was assigned with BLASTP E-value cutoff 1e-5 and C-Score of 0.3 [66]. Tandem duplication is defined for homologous genes no more than 10 genes apart from each other (duplications separated by at least one unrelated gene are further defined as proximal). Syntenic blocks were detected using QUOTA-ALIGN [67] with chaining distance 20. Transitive syntenic relationships (weak secondary syntenic anchoring connected by intermediate homeologs) were identified using an in-house python script. Homeolog groups were formed by single linkage clustering.

The distribution of synonymous substitution rates (Ks) was reported for pairs of homeologs. Pairwise alignment of peptide sequences was produced by CLUSTALW [68] and converted to corresponding DNA alignment using PAL2NAL [69]. Some homeologous gene pairs formed no reliable CLUSTALW alignment for various reasons and were discarded from further analysis. Ks values were calculated using the Nei-Gojobori algorithm [70] implemented in the PAML package [71]. Negative Ks values due to internal error of the PAML package (124 cases), and Ks values greater than 3 (58 cases), which likely reflected saturation of divergence, were discarded. The whole calculation was pipelined in a python script.

**Global gene family classification and phylogenetic analysis**

The complete set of protein coding genes from lotus, sixteen other sequenced angiosperm species (*Arabidopsis thaliana, Carica papaya, Fragaria vesca, Glycine max, Medicago truncatula, Mimulus guttatus, Oryza sativa, Phoenix dactylifera, Populus trichocarpa, Solanum lycopersicum, Solanum tuberosum, Sorghum bicolor, Thellungiella parvula, Theobroma cacao, Vitis vinifera, Zea mays*), and one lycopod (*Selaginella moellendorfii*) were used to identify putative orthologous gene clusters (table S8). Orthogroups were determined using Proteinortho v4.20 [13] using the default settings, except for the minimum algebraic connectivity (-conn=0.05) and the minimum similarity for additional hits (-m=0.75). A total of 529,816 non-redundant genes were classified into 39,649 orthologous gene clusters (orthogroups) containing at least two genes (table S8). Of the 26,685 protein-coding genes in lotus, 21,427 (80.3%) were classified into 10,360 orthogroups, of which 317 contained only lotus genes. The ancestral gene content at key nodes along with the evolutionary changes occurring along the branches leading to these nodes were reconstructed using both parsimony- and likelihood-based approaches, implemented in the program Count [72]. An equal-weight parsimony penalty was used to assess orthogroup gains and losses.

Amino acid alignments for each orthogroup were generated with MAFFT using default parameters. Corresponding DNA sequences were then forced onto the amino acid alignments using custom Perl scripts, and DNA alignments were used in subsequent phylogenetic analysis. Maximum likelihood (ML) analyses were conducted using RAxML version 7.2.1 [73], searching for the best ML tree with the GTRGAMMA model and conducting 100 bootstrap replicates. In total, we constructed 9,502 (out of 10,043) phylogenetics trees. The rest of the orthogroups contained fewer than 4 genes and were not suitable for phylogenetic analysis. Gene family phylogenies were examined for duplications that could have occurred with the gamma duplication in the genomes of core eudicots [7, 23]. Five orthogroups containing MADS box genes were also analyzed using the same methods, but after the inclusion of 34 additional unigenes generated by Vekemans et al (2012).

**Organellar genome assembly and annotation**

The published lotus plastome (NC_015610) was used as a reference for assembly of the lotus plastome using illumina and 454 data. Assembly was done in CLC bio (http://www.clcbio.com/). The sequence depth of the aligned reads averages >78,000 along the entire genome (fig. S13). Annotation was done using DOGMA [74]. The gene map figure was produced using OGDRAW [75]. Gene synteny was determined using *Nicotian tabacum* as a reference [76] because *Nicotiana* is the standard for land plant plastomes. Detailed differences between our newly generated plastome and the lotus released to GenBank were done in Sequencher v. 5.0 (http://genecodes.com/).

Putative mitochondria contigs were extracted from the initial 454 assembly based on coverage (anything with a 40 fold higher coverage than average were investigated) and verified using BLAST alignment to conserved genic sequences. Mitochondria contigs were ordered using 20kb paired-end 454 reads and confirmed using PCR. The draft of the mitochondria genome consists of 21 contigs totaling 453kb. Annotation of the mitochondrial draft assembly was attempted using Mitofy [77]. Mitofy automates the search for known mitochondrial proteins and tRNAs using Blast and tRNAscan-SE to determine the genic content of the genome. Additional work on the draft genome is being done to verify which genes are coding and which are pseudogenes.

**Alternative splicing**

Mapping of ESTs to the corresponding genome sequences and identification of AS isoforms were carried out using ASFinder (http://proteomics.ysu.edu/tools/ASFinder.html/). ASFinder uses SIM4 program to map ESTs to the genome [78]. It then identifies ESTs that are mapped to the same genomic location but have variable exon-intron boundaries as AS isoforms. For genome mapping, the thresholds used included a minimum of 97% identity between aligned ESTs and genomic sequences, a minimum of 80 bp of aligned length, and >85% of EST sequence aligned to the genome. The output of ASFinder was subsequently analyzed and AS events were identified using AStalavista server (http://genome.crg.es/astalavista/) [79].

**Financial support:**

Analyses of the lotus genome are supported by the following sources: Knowledge Innovation Program of the Chinese Academy of Sciences Grant KSCX2-1W-J-20 to YH, Y L, and S L; National Science Foundation (NSF) Plant Genome Program Grant # 0922545 to RM, PM, QY; NSF Plant Genome Program Grant # IOS-1044821 to DRG; NSF: DBI 0849896, MCB 1021718 to AHP; NSF Plant Genome Program Grant #0922742  to CWD; NIH 9R01HG006677-12 to MCS; NIH/NHGRI-R01-HG004694 and NSF IOS-1126998 to MY; National Basic Research Program of China # 2008ZX10002-018, 2010CB945500 and a start-up grant of Fudan University to YZ; NSF MCB 1020458 to BM and BBB; Donald Danforth Plant Science Center startup funds to TCM; National Institutes of Health R37 GM42143 to SSM; Department of Energy DE-FC03-02ER63421 to David Eisenberg; Ruth L. Kirschstein National Research Service Award GM100753 to CEB; ARC Discovery Project Grant No. 0451617 to JW, SAR, KI JSPS; USDA Specific Cooperative Agreement with Hawaii Agriculture Research Center #5320-8-261 to YJZ and MLW; The Ohio Plant Biotechnology Consortium to XM. Grant-in-Aid for Scientific Research (b) Grant No. 24380182  to KI; Hermon Slade Foundation 2009-2011  to SAR, JW; NSF Plant Genome Program Grant IOS-1126998 to M.Y and N.J; and USDA Hatch Grant H862 to REP and NJC. NSF Grant No. IOS-12432275 to DRN.

**References**

1. Zhang H, Zhao X, Ding X, Peterson, AH, Wing RA: **Preparation of megabase-size DNA from plant nuclei.** *Plant J* 1995, 7:175-18.
2. Butler JI, MacCallum I, Kleber M, Shlyakhter IA, Blemonte MK, Lander ES, Musbaum C, Jaffe DB: **ALLPATHS: de novo assembly of whole-genome shotgun microreads.** *Genome Res* 2008, 18:810-20.
3. [Earl D](http://www.ncbi.nlm.nih.gov/pubmed?term=Earl D%5BAuthor%5D&cauthor=true&cauthor_uid=21926179), [Bradnam K](http://www.ncbi.nlm.nih.gov/pubmed?term=Bradnam K%5BAuthor%5D&cauthor=true&cauthor_uid=21926179), [St John J](http://www.ncbi.nlm.nih.gov/pubmed?term=St John J%5BAuthor%5D&cauthor=true&cauthor_uid=21926179), [Darling A](http://www.ncbi.nlm.nih.gov/pubmed?term=Darling A%5BAuthor%5D&cauthor=true&cauthor_uid=21926179), [Lin D](http://www.ncbi.nlm.nih.gov/pubmed?term=Lin D%5BAuthor%5D&cauthor=true&cauthor_uid=21926179), [Fass J](http://www.ncbi.nlm.nih.gov/pubmed?term=Fass J%5BAuthor%5D&cauthor=true&cauthor_uid=21926179), [Yu HO](http://www.ncbi.nlm.nih.gov/pubmed?term=Yu HO%5BAuthor%5D&cauthor=true&cauthor_uid=21926179), [Buffalo V](http://www.ncbi.nlm.nih.gov/pubmed?term=Buffalo V%5BAuthor%5D&cauthor=true&cauthor_uid=21926179), [Zerbino DR](http://www.ncbi.nlm.nih.gov/pubmed?term=Zerbino DR%5BAuthor%5D&cauthor=true&cauthor_uid=21926179), [Diekhans M](http://www.ncbi.nlm.nih.gov/pubmed?term=Diekhans M%5BAuthor%5D&cauthor=true&cauthor_uid=21926179), [Nguyen N](http://www.ncbi.nlm.nih.gov/pubmed?term=Nguyen N%5BAuthor%5D&cauthor=true&cauthor_uid=21926179), [Ariyaratne PN](http://www.ncbi.nlm.nih.gov/pubmed?term=Ariyaratne PN%5BAuthor%5D&cauthor=true&cauthor_uid=21926179), [Sung WK](http://www.ncbi.nlm.nih.gov/pubmed?term=Sung WK%5BAuthor%5D&cauthor=true&cauthor_uid=21926179), [Ning Z](http://www.ncbi.nlm.nih.gov/pubmed?term=Ning Z%5BAuthor%5D&cauthor=true&cauthor_uid=21926179), [Haimel M](http://www.ncbi.nlm.nih.gov/pubmed?term=Haimel M%5BAuthor%5D&cauthor=true&cauthor_uid=21926179), [Simpson JT](http://www.ncbi.nlm.nih.gov/pubmed?term=Simpson JT%5BAuthor%5D&cauthor=true&cauthor_uid=21926179), [Fonseca NA](http://www.ncbi.nlm.nih.gov/pubmed?term=Fonseca NA%5BAuthor%5D&cauthor=true&cauthor_uid=21926179), [Birol İ](http://www.ncbi.nlm.nih.gov/pubmed?term=Birol İ%5BAuthor%5D&cauthor=true&cauthor_uid=21926179), [Docking TR](http://www.ncbi.nlm.nih.gov/pubmed?term=Docking TR%5BAuthor%5D&cauthor=true&cauthor_uid=21926179), [Ho IY](http://www.ncbi.nlm.nih.gov/pubmed?term=Ho IY%5BAuthor%5D&cauthor=true&cauthor_uid=21926179), [Rokhsar DS](http://www.ncbi.nlm.nih.gov/pubmed?term=Rokhsar DS%5BAuthor%5D&cauthor=true&cauthor_uid=21926179), [Chikhi R](http://www.ncbi.nlm.nih.gov/pubmed?term=Chikhi R%5BAuthor%5D&cauthor=true&cauthor_uid=21926179), [Lavenier D](http://www.ncbi.nlm.nih.gov/pubmed?term=Lavenier D%5BAuthor%5D&cauthor=true&cauthor_uid=21926179), [Chapuis G](http://www.ncbi.nlm.nih.gov/pubmed?term=Chapuis G%5BAuthor%5D&cauthor=true&cauthor_uid=21926179), [Naquin D](http://www.ncbi.nlm.nih.gov/pubmed?term=Naquin D%5BAuthor%5D&cauthor=true&cauthor_uid=21926179), [Maillet N](http://www.ncbi.nlm.nih.gov/pubmed?term=Maillet N%5BAuthor%5D&cauthor=true&cauthor_uid=21926179), [Schatz MC](http://www.ncbi.nlm.nih.gov/pubmed?term=Schatz MC%5BAuthor%5D&cauthor=true&cauthor_uid=21926179), [Kelley DR](http://www.ncbi.nlm.nih.gov/pubmed?term=Kelley DR%5BAuthor%5D&cauthor=true&cauthor_uid=21926179), [Phillippy AM](http://www.ncbi.nlm.nih.gov/pubmed?term=Phillippy AM%5BAuthor%5D&cauthor=true&cauthor_uid=21926179), [Koren S](http://www.ncbi.nlm.nih.gov/pubmed?term=Koren S%5BAuthor%5D&cauthor=true&cauthor_uid=21926179), et al: **Assemblathon 1: a competitive assessment of de novo short read assembly methods.** *Genome Res* 2011, 21:2224-41.
4. [Salzberg SL](http://www.ncbi.nlm.nih.gov/pubmed?term=Salzberg SL%5BAuthor%5D&cauthor=true&cauthor_uid=22147368), [Phillippy AM](http://www.ncbi.nlm.nih.gov/pubmed?term=Phillippy AM%5BAuthor%5D&cauthor=true&cauthor_uid=22147368), [Zimin A](http://www.ncbi.nlm.nih.gov/pubmed?term=Zimin A%5BAuthor%5D&cauthor=true&cauthor_uid=22147368), [Puiu D](http://www.ncbi.nlm.nih.gov/pubmed?term=Puiu D%5BAuthor%5D&cauthor=true&cauthor_uid=22147368), [Magoc T](http://www.ncbi.nlm.nih.gov/pubmed?term=Magoc T%5BAuthor%5D&cauthor=true&cauthor_uid=22147368), [Koren S](http://www.ncbi.nlm.nih.gov/pubmed?term=Koren S%5BAuthor%5D&cauthor=true&cauthor_uid=22147368), [Treangen TJ](http://www.ncbi.nlm.nih.gov/pubmed?term=Treangen TJ%5BAuthor%5D&cauthor=true&cauthor_uid=22147368), [Schatz MC](http://www.ncbi.nlm.nih.gov/pubmed?term=Schatz MC%5BAuthor%5D&cauthor=true&cauthor_uid=22147368), [Delcher AL](http://www.ncbi.nlm.nih.gov/pubmed?term=Delcher AL%5BAuthor%5D&cauthor=true&cauthor_uid=22147368), [Roberts M](http://www.ncbi.nlm.nih.gov/pubmed?term=Roberts M%5BAuthor%5D&cauthor=true&cauthor_uid=22147368), [Marçais G](http://www.ncbi.nlm.nih.gov/pubmed?term=Marçais G%5BAuthor%5D&cauthor=true&cauthor_uid=22147368), [Pop M](http://www.ncbi.nlm.nih.gov/pubmed?term=Pop M%5BAuthor%5D&cauthor=true&cauthor_uid=22147368), [Yorke JA](http://www.ncbi.nlm.nih.gov/pubmed?term=Yorke JA%5BAuthor%5D&cauthor=true&cauthor_uid=22147368): **GAGE: A critical evaluation of genome assemblies and assembly algorithms.** *Genome Res* 2012, **22**:557-67.
5. [Schatz MC](http://www.ncbi.nlm.nih.gov/pubmed?term=Schatz MC%5BAuthor%5D&cauthor=true&cauthor_uid=22199379), [Phillippy AM](http://www.ncbi.nlm.nih.gov/pubmed?term=Phillippy AM%5BAuthor%5D&cauthor=true&cauthor_uid=22199379), [Sommer DD](http://www.ncbi.nlm.nih.gov/pubmed?term=Sommer DD%5BAuthor%5D&cauthor=true&cauthor_uid=22199379), [Delcher AL](http://www.ncbi.nlm.nih.gov/pubmed?term=Delcher AL%5BAuthor%5D&cauthor=true&cauthor_uid=22199379), [Puiu D](http://www.ncbi.nlm.nih.gov/pubmed?term=Puiu D%5BAuthor%5D&cauthor=true&cauthor_uid=22199379), [Narzisi G](http://www.ncbi.nlm.nih.gov/pubmed?term=Narzisi G%5BAuthor%5D&cauthor=true&cauthor_uid=22199379), [Salzberg SL](http://www.ncbi.nlm.nih.gov/pubmed?term=Salzberg SL%5BAuthor%5D&cauthor=true&cauthor_uid=22199379), [Pop M](http://www.ncbi.nlm.nih.gov/pubmed?term=Pop M%5BAuthor%5D&cauthor=true&cauthor_uid=22199379): **Hawkeye and AMOS: visualizing and assessing the quality of genome assemblies.** *Briefings in bioinformatics* 2011*,* doi: 10.1093/bib/bbr074.
6. Li H, Durbin R: **Fast and accurate short read alignment with Burrows-Wheeler transform.** *Bioinformatics* 2009, **25**:1754-60.
7. Rice P, Longden I, Bleasby A: **EMBOSS: the European Molecular Biology Open Software Suite.** *Trends Genet* 2000, **16**:276-277.
8. [Chevreux B](http://www.ncbi.nlm.nih.gov/pubmed?term=Chevreux B%5BAuthor%5D&cauthor=true&cauthor_uid=15140833), [Pfisterer T](http://www.ncbi.nlm.nih.gov/pubmed?term=Pfisterer T%5BAuthor%5D&cauthor=true&cauthor_uid=15140833), [Drescher B](http://www.ncbi.nlm.nih.gov/pubmed?term=Drescher B%5BAuthor%5D&cauthor=true&cauthor_uid=15140833), [Driesel AJ](http://www.ncbi.nlm.nih.gov/pubmed?term=Driesel AJ%5BAuthor%5D&cauthor=true&cauthor_uid=15140833), [Müller WE](http://www.ncbi.nlm.nih.gov/pubmed?term=Müller WE%5BAuthor%5D&cauthor=true&cauthor_uid=15140833), [Wetter T](http://www.ncbi.nlm.nih.gov/pubmed?term=Wetter T%5BAuthor%5D&cauthor=true&cauthor_uid=15140833), [Suhai S](http://www.ncbi.nlm.nih.gov/pubmed?term=Suhai S%5BAuthor%5D&cauthor=true&cauthor_uid=15140833): **Using the miraEST assembler for reliable and automated mRNA transcript assembly and SNP detection in sequenced ESTs**. *Genome Res* 2004, **14**:1147-1159.
9. Huang X, Madan A: **CAP3: A DNA sequence assembly program.** *Genome Res* 1999, 9: 868-877.
10. [Yu Q](http://www.ncbi.nlm.nih.gov/pubmed?term=Yu Q%5BAuthor%5D&cauthor=true&cauthor_uid=16117847), [Moore PH](http://www.ncbi.nlm.nih.gov/pubmed?term=Moore PH%5BAuthor%5D&cauthor=true&cauthor_uid=16117847), [Albert HH](http://www.ncbi.nlm.nih.gov/pubmed?term=Albert HH%5BAuthor%5D&cauthor=true&cauthor_uid=16117847), [Roader AH](http://www.ncbi.nlm.nih.gov/pubmed?term=Roader AH%5BAuthor%5D&cauthor=true&cauthor_uid=16117847), [Ming R](http://www.ncbi.nlm.nih.gov/pubmed?term=Ming R%5BAuthor%5D&cauthor=true&cauthor_uid=16117847): **Cloning and characterization of a FLORICAULA/LEAFY ortholog, PFL, in polygamous papaya.** *Cell Research* 2005, **15**:576-84.
11. Holt C, Yandell M: **MAKER2: an annotation pipeline and genome-database management tool for second-generation genome projects.** *BMC Bioinformatics* 2011, **12**:491.
12. Jurka J, Kapitonov VV, Pavlicek A, Klonowski P, Kohany O, Walichiewicz J: **Repbase Update, a database of eukaryotic repetitive elements.** *Cytogentic and Genome Research* 2005, **110**:462-467.
13. Korf I, Yandell M, Bedel J: *BLAST* O’reily, Cambridge, 2003, 81.
14. [Cantarel BL](http://www.ncbi.nlm.nih.gov/pubmed?term=Cantarel BL%5BAuthor%5D&cauthor=true&cauthor_uid=18025269), [Korf I](http://www.ncbi.nlm.nih.gov/pubmed?term=Korf I%5BAuthor%5D&cauthor=true&cauthor_uid=18025269), [Robb SM](http://www.ncbi.nlm.nih.gov/pubmed?term=Robb SM%5BAuthor%5D&cauthor=true&cauthor_uid=18025269), [Parra G](http://www.ncbi.nlm.nih.gov/pubmed?term=Parra G%5BAuthor%5D&cauthor=true&cauthor_uid=18025269), [Ross E](http://www.ncbi.nlm.nih.gov/pubmed?term=Ross E%5BAuthor%5D&cauthor=true&cauthor_uid=18025269), [Moore B](http://www.ncbi.nlm.nih.gov/pubmed?term=Moore B%5BAuthor%5D&cauthor=true&cauthor_uid=18025269), [Holt C](http://www.ncbi.nlm.nih.gov/pubmed?term=Holt C%5BAuthor%5D&cauthor=true&cauthor_uid=18025269), [Sánchez Alvarado A](http://www.ncbi.nlm.nih.gov/pubmed?term=Sánchez Alvarado A%5BAuthor%5D&cauthor=true&cauthor_uid=18025269), [Yandell M](http://www.ncbi.nlm.nih.gov/pubmed?term=Yandell M%5BAuthor%5D&cauthor=true&cauthor_uid=18025269): **MAKER: an easy-to-use annotation pipeline designed for emerging model organism genomes.** *Genome Res* 2008, **18**:188-96.
15. Korf I: **Gene Finding In Novel Genomes.** *BMC Bioinformatics* 2004*,* **5**:59.
16. Stanke M, Waack S: **Gene prediction with a hidden Markov model and a new intron submodel**. *Bioinformatics* 2003, **2**:215-225.
17. Stanke M, Diekhans M, Baertsch R, Haussler : **Using native and syntenically mapped cDNA alignments to improve de novo gene finding.** *Bioinformatics* 2008, **24**:637-44.
18. [Quevillon E](http://www.ncbi.nlm.nih.gov/pubmed?term=Quevillon E%5BAuthor%5D&cauthor=true&cauthor_uid=15980438), [Silventoinen V](http://www.ncbi.nlm.nih.gov/pubmed?term=Silventoinen V%5BAuthor%5D&cauthor=true&cauthor_uid=15980438), [Pillai S](http://www.ncbi.nlm.nih.gov/pubmed?term=Pillai S%5BAuthor%5D&cauthor=true&cauthor_uid=15980438), [Harte N](http://www.ncbi.nlm.nih.gov/pubmed?term=Harte N%5BAuthor%5D&cauthor=true&cauthor_uid=15980438), [Mulder N](http://www.ncbi.nlm.nih.gov/pubmed?term=Mulder N%5BAuthor%5D&cauthor=true&cauthor_uid=15980438), [Apweiler R](http://www.ncbi.nlm.nih.gov/pubmed?term=Apweiler R%5BAuthor%5D&cauthor=true&cauthor_uid=15980438), [Lopez R](http://www.ncbi.nlm.nih.gov/pubmed?term=Lopez R%5BAuthor%5D&cauthor=true&cauthor_uid=15980438): **InterProScan: protein domains identifier.** *Nucleic Acids Res 2005,* **33**:116-20.
19. Altschul SF, Gish W, Miller W, Myers E-W, Lipman DJ: **Basic local alignment search tool.** *J Mol Biol* 1990, **5**:403-10.
20. Mortazavi A, Williams BA, McCue K, Schaeffer L, Wold B: **Mapping and quantifying mammalian transcriptomes by RNA-Seq.** *Nat Methods* 2008, **5**:621-628.
21. [Gan Q](http://www.ncbi.nlm.nih.gov/pubmed?term=Gan Q%5BAuthor%5D&cauthor=true&cauthor_uid=20398323), [Schones DE](http://www.ncbi.nlm.nih.gov/pubmed?term=Schones DE%5BAuthor%5D&cauthor=true&cauthor_uid=20398323), [Ho Eun S](http://www.ncbi.nlm.nih.gov/pubmed?term=Ho Eun S%5BAuthor%5D&cauthor=true&cauthor_uid=20398323), [Wei G](http://www.ncbi.nlm.nih.gov/pubmed?term=Wei G%5BAuthor%5D&cauthor=true&cauthor_uid=20398323), [Cui K](http://www.ncbi.nlm.nih.gov/pubmed?term=Cui K%5BAuthor%5D&cauthor=true&cauthor_uid=20398323), [Zhao K](http://www.ncbi.nlm.nih.gov/pubmed?term=Zhao K%5BAuthor%5D&cauthor=true&cauthor_uid=20398323), [Chen X](http://www.ncbi.nlm.nih.gov/pubmed?term=Chen X%5BAuthor%5D&cauthor=true&cauthor_uid=20398323)*:* **Monovalent and unpoised status of most genes in undifferentiated cell-enriched Drosophila testis.** *Genome Biol* 2010, 11, doi:Artn R42 Doi 10.1186/Gb-2010-11-4-R42.
22. Ellinghaus D, Kurtz S, Willhoeft U: **LTR harvest, an efficient and flexible software for de novo detection of LTR retrotransposons.** *BMC Bioinformatics* 2008, **9**:18.
23. [Steinbiss S](http://www.ncbi.nlm.nih.gov/pubmed?term=Steinbiss S%5BAuthor%5D&cauthor=true&cauthor_uid=19786494), [Willhoeft U](http://www.ncbi.nlm.nih.gov/pubmed?term=Willhoeft U%5BAuthor%5D&cauthor=true&cauthor_uid=19786494), [Gremme G](http://www.ncbi.nlm.nih.gov/pubmed?term=Gremme G%5BAuthor%5D&cauthor=true&cauthor_uid=19786494), [Kurtz S](http://www.ncbi.nlm.nih.gov/pubmed?term=Kurtz S%5BAuthor%5D&cauthor=true&cauthor_uid=19786494): **Fine-grained annotation and classification of de novo predicted LTRretrotransposons.** *Nucleic Acids Res* 2009, **37**:7002-13.
24. Morgenstern B: **DIALIGN: multiple DNA and protein sequence alignment at BiBiServ.** N*ucleic Acids Res* 2004, **32**:33-36.
25. Han YS, Wessler R: **MITE-Hunter: a program for discovering miniature inverted-repeat transposable elements from genomic sequences.** *Nucleic Acids Res* 2010*,* **38**:199.
26. Ferguson AA, Jiang N: **Mutator-like elements with multiple long terminal inverted repeats in plants**. *Comp Funct Genomics* 2012, **2012**:14.
27. Wicker T, Sabot F, Hua-Van A, Bennetzen JL, Capy P, Chalhoub B, Flavell A, Leroy P, Morgante M, Panaud O, Paux E, SanMiguel P, Schulman AH: **A unified classification system for eukaryotic transposable elements**. *Nat Rev Genet* 2007, **8**:973-982.
28. Hanada K, [Vallejo V](http://www.ncbi.nlm.nih.gov/pubmed?term=Vallejo V%5BAuthor%5D&cauthor=true&cauthor_uid=19136648), [Nobuta K](http://www.ncbi.nlm.nih.gov/pubmed?term=Nobuta K%5BAuthor%5D&cauthor=true&cauthor_uid=19136648), [Slotkin RK](http://www.ncbi.nlm.nih.gov/pubmed?term=Slotkin RK%5BAuthor%5D&cauthor=true&cauthor_uid=19136648), [Lisch D](http://www.ncbi.nlm.nih.gov/pubmed?term=Lisch D%5BAuthor%5D&cauthor=true&cauthor_uid=19136648), [Meyers BC](http://www.ncbi.nlm.nih.gov/pubmed?term=Meyers BC%5BAuthor%5D&cauthor=true&cauthor_uid=19136648), [Shiu SH](http://www.ncbi.nlm.nih.gov/pubmed?term=Shiu SH%5BAuthor%5D&cauthor=true&cauthor_uid=19136648), [Jiang N](http://www.ncbi.nlm.nih.gov/pubmed?term=Jiang N%5BAuthor%5D&cauthor=true&cauthor_uid=19136648): **The functional role of pack-MULEs in rice inferred from purifying selection and expression profile.** *Plant Cell* 2009, **21**:25-38.

| 1. Du C, Caronna J, He L, Dooner HK: **Computational prediction and molecular confirmation of *Helitron* transposons in the maize genome.** *BMC Genomics* 2008, **9**:51. |
| --- |
| 1. Du C, Fefelova N, Caronna J, He L, Dooner, HK: **The polychromatic Helitron landscape of the maize genome.** *Proc Natl Acad Sci USA* 2009, **106**:19747. |

1. Xiong W, Li T, Chen K, Tang K: **Local combinational variables: an approach used in DNA-binding helix-turn-helix motif prediction with sequence information.** *Nucleic Acids Res* 2009, **37**:5632.
2. Kielbasa SM, Wan R, Sato K, Horton P, Frith MC: **Adaptive seeds tame genomic sequence comparison.** *Genome Res* 2011, **21**:487.
3. Putnam NH, Butts T, Ferrier DE, Furlong RF, Hellsten U, Kawashima T, Robinson-Rechavi M, Shoguchi E, Terry A, Yu JK, Benito-Gutiérrez EL, Dubchak I, Garcia-Fernàndez J, Gibson-Brown JJ, Grigoriev IV, Horton AC, de Jong PJ, Jurka J, Kapitonov VV, Kohara Y, Kuroki Y, Lindquist E, Lucas S, Osoegawa K, Pennacchio LA, Salamov AA, Satou Y, Sauka-Spengler T, Schmutz J, Shin-I T, et al: **The amphioxus genome and the evolution of the chordate karyotype.** *Nature* 2008, **453**:1064.
4. Tang H, [Lyons E](http://www.ncbi.nlm.nih.gov/pubmed?term=Lyons E%5BAuthor%5D&cauthor=true&cauthor_uid=21501495), [Pedersen B](http://www.ncbi.nlm.nih.gov/pubmed?term=Pedersen B%5BAuthor%5D&cauthor=true&cauthor_uid=21501495), [Schnable JC](http://www.ncbi.nlm.nih.gov/pubmed?term=Schnable JC%5BAuthor%5D&cauthor=true&cauthor_uid=21501495), [Paterson AH](http://www.ncbi.nlm.nih.gov/pubmed?term=Paterson AH%5BAuthor%5D&cauthor=true&cauthor_uid=21501495), [Freeling M](http://www.ncbi.nlm.nih.gov/pubmed?term=Freeling M%5BAuthor%5D&cauthor=true&cauthor_uid=21501495): **Screening synteny blocks in pairwise genome comparisons through integer programming.** *BMC Bioinformatics* 2011, **12**:102.
5. Thompson JD, Higgins DG, Gibson TJ: **CLUSTALW: improving the sensitivity of progressive multiple sequence alignment through sequence weighting, position-specific gap penalties and weight matrix choice.** *Nucleic Acids Res* 1994, **22**:4673.
6. Suyama M, Torrents D, Bork P: **PAL2NAL: robust conversion of protein sequence alignments into the corresponding codon alignments.** *Nucleic Acids Res* 2006, **34**:609.
7. Nei M, Gojobori, T: **Simple methods for estimating the numbers of synonymous and nonsynonymous nucleotide substitutions.** *Mol Biol Evol* 1986, **3**:418.
8. Yang ZH: **PAML: a program package for phylogenetic analysis by maximum likelihood.** *Computer Applications in the Biosciences* 1997, **13**:555.
9. Csurös M: **Count: evolutionary analysis of phylogenetic profiles with parsimony and likelihood.** *Bioinformatics* 2010, **26**:1910-12.
10. Stamatakis A: **RAxML-VI-HPC: maximum likelihood-based phylogenetic analyses with thousands of taxa and mixed models.** *Bioinformatics* 2006, **22**:2688-90.
11. Wyman S-K, Jansen RK, Boore L: **Automatic annotation of organellar genomes with DOGMA.** *Bioinformatics* 2004, **20**:3252-55.
12. Lohse M, Drechsel O, Bock R: **OrganellarGenomeDRAW (OGDRAW) - a tool for the easy generation of high-quality custom graphical maps of plastid and mitochondrial genomes.** *Current Genetics* 2007, **52**:267-74.
13. [Shinozaki K](http://www.ncbi.nlm.nih.gov/pubmed?term=Shinozaki K%5BAuthor%5D&cauthor=true&cauthor_uid=16453699), [Ohme M](http://www.ncbi.nlm.nih.gov/pubmed?term=Ohme M%5BAuthor%5D&cauthor=true&cauthor_uid=16453699), [Tanaka M](http://www.ncbi.nlm.nih.gov/pubmed?term=Tanaka M%5BAuthor%5D&cauthor=true&cauthor_uid=16453699), [Wakasugi T](http://www.ncbi.nlm.nih.gov/pubmed?term=Wakasugi T%5BAuthor%5D&cauthor=true&cauthor_uid=16453699), [Hayashida N](http://www.ncbi.nlm.nih.gov/pubmed?term=Hayashida N%5BAuthor%5D&cauthor=true&cauthor_uid=16453699), [Matsubayashi T](http://www.ncbi.nlm.nih.gov/pubmed?term=Matsubayashi T%5BAuthor%5D&cauthor=true&cauthor_uid=16453699), [Zaita N](http://www.ncbi.nlm.nih.gov/pubmed?term=Zaita N%5BAuthor%5D&cauthor=true&cauthor_uid=16453699), [Chunwongse J](http://www.ncbi.nlm.nih.gov/pubmed?term=Chunwongse J%5BAuthor%5D&cauthor=true&cauthor_uid=16453699), [Obokata J](http://www.ncbi.nlm.nih.gov/pubmed?term=Obokata J%5BAuthor%5D&cauthor=true&cauthor_uid=16453699), [Yamaguchi-Shinozaki K](http://www.ncbi.nlm.nih.gov/pubmed?term=Yamaguchi-Shinozaki K%5BAuthor%5D&cauthor=true&cauthor_uid=16453699), [Ohto C](http://www.ncbi.nlm.nih.gov/pubmed?term=Ohto C%5BAuthor%5D&cauthor=true&cauthor_uid=16453699), [Torazawa K](http://www.ncbi.nlm.nih.gov/pubmed?term=Torazawa K%5BAuthor%5D&cauthor=true&cauthor_uid=16453699), [Meng BY](http://www.ncbi.nlm.nih.gov/pubmed?term=Meng BY%5BAuthor%5D&cauthor=true&cauthor_uid=16453699), [Sugita M](http://www.ncbi.nlm.nih.gov/pubmed?term=Sugita M%5BAuthor%5D&cauthor=true&cauthor_uid=16453699), [Deno H](http://www.ncbi.nlm.nih.gov/pubmed?term=Deno H%5BAuthor%5D&cauthor=true&cauthor_uid=16453699), [Kamogashira T](http://www.ncbi.nlm.nih.gov/pubmed?term=Kamogashira T%5BAuthor%5D&cauthor=true&cauthor_uid=16453699), [Yamada K](http://www.ncbi.nlm.nih.gov/pubmed?term=Yamada K%5BAuthor%5D&cauthor=true&cauthor_uid=16453699), [Kusuda J](http://www.ncbi.nlm.nih.gov/pubmed?term=Kusuda J%5BAuthor%5D&cauthor=true&cauthor_uid=16453699), [Takaiwa F](http://www.ncbi.nlm.nih.gov/pubmed?term=Takaiwa F%5BAuthor%5D&cauthor=true&cauthor_uid=16453699), [Kato A](http://www.ncbi.nlm.nih.gov/pubmed?term=Kato A%5BAuthor%5D&cauthor=true&cauthor_uid=16453699), [Tohdoh N](http://www.ncbi.nlm.nih.gov/pubmed?term=Tohdoh N%5BAuthor%5D&cauthor=true&cauthor_uid=16453699), [Shimada H](http://www.ncbi.nlm.nih.gov/pubmed?term=Shimada H%5BAuthor%5D&cauthor=true&cauthor_uid=16453699), [Sugiura M](http://www.ncbi.nlm.nih.gov/pubmed?term=Sugiura M%5BAuthor%5D&cauthor=true&cauthor_uid=16453699): **The complete nucleotide sequence of the tobacco chloroplast genome: its gene organization and expression.** *The EMBO Journal* 1986, **5**:2043-49.
14. Alverson AJ, [Wei X](http://www.ncbi.nlm.nih.gov/pubmed?term=Wei X%5BAuthor%5D&cauthor=true&cauthor_uid=20118192), [Rice DW](http://www.ncbi.nlm.nih.gov/pubmed?term=Rice DW%5BAuthor%5D&cauthor=true&cauthor_uid=20118192), [Stern DB](http://www.ncbi.nlm.nih.gov/pubmed?term=Stern DB%5BAuthor%5D&cauthor=true&cauthor_uid=20118192), [Barry K](http://www.ncbi.nlm.nih.gov/pubmed?term=Barry K%5BAuthor%5D&cauthor=true&cauthor_uid=20118192), [Palmer JD](http://www.ncbi.nlm.nih.gov/pubmed?term=Palmer JD%5BAuthor%5D&cauthor=true&cauthor_uid=20118192): **Insights into the evolution of mitochondrial genome size from complete sequences of *Citrullus lanatus* and *Cucurbita pepo* (Cucurbitaceae).** *Mol Biol Evol* 2010, **27**:1436-48.
15. Florea L, Hartzell G, Zhang Z, Rubin G-M, Miller W: **A computer program for aligning a cDNA sequence with a genomic DNA sequence.** *Genome Res1998,* **8**:967-74.
16. Foissac S, Sammeth M: **ASTALAVISTA: dynamic and flexible analysis of alternative splicing events in custom gene datasets.** *Nucleic Acids Res* 2007, **35**:297–99.
17. APG II. **An update of the angiosperm phylogeny group classification for the orders and families of flowering plants: APG II.** *Bot J Linn Soc* 2003, **141**:399-36.

**Table S1.** Summary of genome assembly and annotation of ‘China Antique’.

| (a) Assembly | Status | Number | N50 (kb) | Longest (kb) | size (Mb) | % assembly |
| --- | --- | --- | --- | --- | --- | --- |
| Contigs | All | 58409 | 38.8 | 286 | 707 | 76.1 |
| Scaffold | All | 3605 | 3,435 | 14,300 | 804 | 86.5 |
| (b) Annotation | number | Average size (bp) | Median size (bp) | Total Length (Mb) | % of genome | % GC |
| Gene | 26,685 | 6562 | 3917 | 175 | 21.7 | 36 |
| Exons | 132,653 | 294 | 153 | 39 | 4.8 | 43 |
| Introns | 108,887 | 1249 | 283 | 136 | 16.9 | 34 |
| miRNA | 160 | 140 | 150 | 0.0237 | 3.11E-06 | 46.7 |
| tRNA | 960 | 114 | 113 | 0.1092 | 1.43E-05 | 46.5 |

**Table S2.** Assembly statistics of 35 sequenced plant genomes.

| | common name | scientififc name | year | size (Mb) | assem (Mb) | gene (#) | contig N50 (kb) | scaffold N50 (kb) | | --- | --- | --- | --- | --- | --- | --- | --- | | arabidopsis | *Arabidopsis thaliana* | 2000 | 125 | 115 | 25,498 | NA | NA | | rice | *Oryza sativa* | 2002 | 430 | 362 | 59,855 | 7 | 12 | | rice | *Oryza sativa* | 2002 | 420 | 389 | 29,961 | NA | NA | | rice | *Oryza sativa* | 2005 | 403 | 388.8 | 37,544 | NA | NA | | Black Cottonwood | *Populus trichocarpa* | 2006 | 485 | 410 | 45,555 | 126 | 3,100 | | grape | *Vitis vinifera* | 2007 | 475 | 487.1 | 30,434 | 66 | 2,065 | | moss | *Physcomitrella patens* | 2008 | 510 | 480 | 35,938 | 292 | 1,320 | | grape | *Vitis vinifera* | 2007 | 504.6 | 477.1 | 29,585 | 18 | 1,330 | | papaya | *Carica papaya* | 2008 | 372 | 370 | 28,629 | 11 | 1,000 | | lotus | *Lotus japonicus* | 2008 | 472 | 315 | 30,799 | NA | NA | | sorghum | *Sorghum bicolor* | 2009 | 818 | 738.5 | 34,496 | 195 | 62,400 | | cucumber | *Cucumis sativus* | 2009 | 367 | 243.5 | 26,682 | 20 | 1,140 | | Maize | *Zea mays* | 2009 | 2300 | 2048 | 32,540 | 40 | 76 | | soybean | *Glycine max* | 2010 | 1115 | 973.3 | 46,430 | 189 | 47,800 | | brachypodium | *Brachypodium distachyon* | 2010 | 272 | 272 | 25,532 | 348 | 59,300 | | castor bean | *Ricinus communis* | 2010 | 320 | 325.5 | 31,237 | 21 | 561 | | apple | *Malus x domestica* | 2010 | 742.3 | 603.9 | 57,386 | 13 | 1,542 | | jatropha | *Jatropha curcas* | 2010 | 380 | 285.8 | 40,929 | 4 | NA | | cocoa | *Theobroma cacao* | 2011 | 430 | 326.9 | 28,798 | 20 | 473 | | strawberry | *Fragaria vesca* | 2011 | 240 | 209.8 | 34,809 | NA | 1,300 | | Lyrata | *Arabidopsis lyrata* | 2011 | 207 | 206.7 | 32,670 | 227 | 24,500 | | spikemoss | *Selaginella moellendorffii* | 2011 | 110 | 212.6 | 22,285 | 120 | 1,700 | | date palm | *Phoenix dactylifera* | 2011 | 658 | 381 | 28,890 | 6 | 30 | | potato | *Solanum tuberosum* | 2011 | 844 | 727 | 39,031 | 31 | 1,318 | | Thellungiella | *Thellungiella parvula* | 2011 | 140 | 137.09 | 30,419 | NA | 5,290 | | cucumber | *Cucumis sativus* | 2011 | 367 | 322 | 26,587 | 23 | 319 | | Chinese cabbage | *Brassica rapa* | 2011 | 485 | 283.8 | 41,174 | 27 | 1,971 | | hemp | *Cannabis sativa* | 2011 | 820 | 786.6 | 30,074 | 2 | 16 | | pigeon pea | *Cajanus cajan* | 2012 | 833 | 605 | 48,680 | 22 | 516 | | medicago | *Medicago truncatula* | 2011 | 454 | 262.43 | 62,388 | NA | 1,270 | | setaria | *Setaria italica* | 2012 | 490 | 423 | 38,801 | 25 | 1,007 | | setaria | *Setaria indica* | 2012 | 510 | 396.7 | 35,471 | 126 | 47,300 | | tomato | *Solanum lycopersicum* | 2012 | 900 | 760 | 34,727 | 87 | 16,467 | | melon | *Cucumis melo* | 2012 | 450 | 375 | 27,427 | 18 | 4,680 | | Banana | *Musa acuminata malaccensis* | 2012 | 523 | 472 | 36,542 | 43 | 1,311 | | Wheat | *Triticum aestivum* |  | 17,000 | 0 | 94,000 | NA | NA | | Barely | *Hordeum vulgare* L. | 2012 | 5,100 | 4,560 | 79,379 | 904 | NA | | Orange | *Citrus sinensis* | 2012 | 367 | 320.5 | 29,445 | 50 | 1,690 | | Watermelon | *Citrullus lanatus* | 2012 | 425 | 353.5 | 23,440 | 26.38 | 2,380 | | **Sacred Lotus** | ***Nelumbo nucifera*** | **TBD** | **929** | **804** | **26,685** | **39** | **3,435** |   ***** This is a briviated version, and the full version of Table S1 is in a separate Excel file.   | Abreviations | | --- | | NA, not available or not reported in the primary publication | | Assembled genome size is based on scaffold (super-scaffold) when available and not anchored scaffolds | | Repeat estimate varies by genome paper; total repeat % or TE% is taken when reported | | TBD, to be determined |   **Table S3**. American lotus genetic map, RAD markers (repeat masked genome as reference sequence) and SSRs | | | | | | | | | | |  |
| --- | --- | --- | --- | --- | --- | --- | --- | --- | --- | --- | --- | --- | --- | --- | --- | --- | --- | --- | --- | --- | --- | --- | --- | --- | --- | --- | --- | --- | --- | --- | --- | --- | --- | --- | --- | --- | --- | --- | --- | --- | --- | --- | --- | --- | --- | --- | --- | --- | --- | --- | --- | --- | --- | --- | --- | --- | --- | --- | --- | --- | --- | --- | --- | --- | --- | --- | --- | --- | --- | --- | --- | --- | --- | --- | --- | --- | --- | --- | --- | --- | --- | --- | --- | --- | --- | --- | --- | --- | --- | --- | --- | --- | --- | --- | --- | --- | --- | --- | --- | --- | --- | --- | --- | --- | --- | --- | --- | --- | --- | --- | --- | --- | --- | --- | --- | --- | --- | --- | --- | --- | --- | --- | --- | --- | --- | --- | --- | --- | --- | --- | --- | --- | --- | --- | --- | --- | --- | --- | --- | --- | --- | --- | --- | --- | --- | --- | --- | --- | --- | --- | --- | --- | --- | --- | --- | --- | --- | --- | --- | --- | --- | --- | --- | --- | --- | --- | --- | --- | --- | --- | --- | --- | --- | --- | --- | --- | --- | --- | --- | --- | --- | --- | --- | --- | --- | --- | --- | --- | --- | --- | --- | --- | --- | --- | --- | --- | --- | --- | --- | --- | --- | --- | --- | --- | --- | --- | --- | --- | --- | --- | --- | --- | --- | --- | --- | --- | --- | --- | --- | --- | --- | --- | --- | --- | --- | --- | --- | --- | --- | --- | --- | --- | --- | --- | --- | --- | --- | --- | --- | --- | --- | --- | --- | --- | --- | --- | --- | --- | --- | --- | --- | --- | --- | --- | --- | --- | --- | --- | --- | --- | --- | --- | --- | --- | --- | --- | --- | --- | --- | --- | --- | --- | --- | --- | --- | --- | --- | --- | --- | --- | --- | --- | --- | --- | --- | --- | --- | --- | --- | --- | --- | --- | --- | --- | --- | --- | --- | --- | --- | --- | --- | --- | --- | --- | --- | --- | --- | --- | --- | --- | --- | --- | --- | --- | --- | --- | --- | --- | --- | --- | --- | --- | --- | --- | --- | --- | --- | --- | --- | --- | --- | --- | --- | --- | --- | --- | --- | --- | --- | --- | --- | --- | --- | --- |
|  | | |  | |  |  | |  | | |  |
| Linkage group | Distance(cM) | Bin Markers or SSR | | Bin Markers or SSRs per cM | | | SSRs | | RAD Bin Markers | Involved RAD markers | |
| LG1 | 97.7 | 203 | | 2.1 | | | 39 | | 164 | 999 | |
| LG2 | 75.8 | 79 | | 1 | | | 16 | | 63 | 372 | |
| LG3 | 69.1 | 114 | | 1.6 | | | 14 | | 100 | 688 | |
| LG4 | 58.3 | 83 | | 1.4 | | | 14 | | 69 | 451 | |
| LG5 | 51.1 | 61 | | 1.2 | | | 18 | | 43 | 249 | |
| LG6 | 48.2 | 80 | | 1.7 | | | 19 | | 61 | 496 | |
| LG7 | 44.9 | 33 | | 0.7 | | | 3 | | 30 | 243 | |
| LG8 | 27.7 | 32 | | 1.2 | | | 10 | | 22 | 220 | |
| LG9 | 21.5 | 13 | | 0.6 | | | 3 | | 10 | 177 | |
| Total | 494.3 | 698 | | 1.41 | | | 136 | | 562 | 3895 | |

**Table S4.** Transposable elements and other repetitive sequences in the assembled fraction of the lotus genome

| Class | Sub-class | Superfamily | Copy number*  (x1000) | Fraction of genome (%) |
| --- | --- | --- | --- | --- |
| Class I | LTR Retrotransposon | LTR/*Copia* | 47.5 | 11.9 |
|  |  | LTR/*Gypsy* | 49.6 | 11.8 |
|  |  | LTR/Unknown | 14.1 | 1.4 |
|  |  | Total LTR | 111.2 | 25.1 |
|  | Non-LTR Retrotranspson | LINE | 28.8. | 6.3 |
|  |  | SINE | 4.2 | 0.1 |
|  |  | Total non-LTR | 33.0 | 6.4 |
|  | Total Class I | | 144.2 | 31.5 |
| Class II |  | CACTA | 4.8 | 0.4 |
|  |  | *hAT* | 103.9 | 6.8 |
|  |  | MULE | 58.9 | 2.5 |
|  |  | *PIF*/*Tourist* | 65.5 | 2.7 |
|  |  | *Helitron* | 16.5 | 3.6 |
|  |  | DNA/Unknown | 2.2 | 0.2 |
|  | Total Class II | | 251.8 | 16.2 |
| Total transposable elements | | | 396.0 | 47.7 |
| Unknown repeats | | | 232.2 | 8.9 |
| Total repeats | | | 628.2 | 56.6 |

*Copy number includes fragmented copies.

**Table S5.** Distribution of alternative splicing events.

| **Alternative splicing (AS) type** | **Number** | **Percentage of total AS** |
| --- | --- | --- |
| Intron retention | 109 | 62.6 |
| Alternative donor sites | 14 | 8 |
| Alternative acceptor sites | 13 | 7.5 |
| Exon skipping | 7 | 4 |
| Others | 31 | 17.8 |
| **Total** | **174** |  |

**Table S6** RPKM values of Rhizome specific genes

(It is a large Excel file and will be send separately

**Table S7.** Inferred minimum gene set. Calculated using the smallest observed gene counts for each orthogroup in restricted subsets of the taxa, and summed across all of the orthogroups. Increases in gene numbers are suggested through eudicot and monocot history.

| **Lineage** | **Minimum number of genes** | **Minimum number of orthogroups** |
| --- | --- | --- |
| Tracheophytes | 4223 | 2919 |
| Eudicots+Monocots | 6423 | 4095 |
| Eudicots | 7165 | 4585 |
| Core-Eudicots | 7559 | 4798 |
| Rosids | 8404 | 5403 |
| Asterids | 16006 | 8136 |
| Monocots | 11645 | 6537 |
| Grass | 19575 | 9106 |

**Table S8.** Wagner parsimony ancestral orthogroup reconstruction using equal gain-loss penalty, as implemented in the program Count (Csurös 2010). Classification information for *Mimulus guttatus* and *Solanum lycopersicum* has been masked from this table to respect pre-publication data release restrictions.

| **TAXON/NODE** | **SINGLE** | **MULTI** | **GAIN** | **LOSS** | **EXPANSION** | **CONTRACTION** |
| --- | --- | --- | --- | --- | --- | --- |
| *Solanum tuberosum* | 14282 | 9190 | 3933 | 881 | 2107 | 192 |
| *Arabidopsis thaliana* | 10667 | 5290 | 690 | 141 | 529 | 48 |
| *Thellungiella parvula* | 10852 | 5279 | 928 | 194 | 389 | 85 |
| *Carica papaya* | 10991 | 3991 | 2228 | 915 | 278 | 539 |
| *Theobroma cacao* | 13318 | 6742 | 3636 | 140 | 511 | 98 |
| *Populus trichocarpa* | 12355 | 8569 | 2477 | 108 | 3187 | 43 |
| *Fragaria vesca* | 11251 | 5248 | 2161 | 638 | 303 | 379 |
| *Medicago truncatula* | 12399 | 7688 | 4122 | 1764 | 312 | 672 |
| *Glycine max* | 11638 | 9700 | 1814 | 217 | 3684 | 42 |
| *Vitis vinifera* | 10408 | 4317 | 1499 | 714 | 482 | 340 |
| ***Nelumbo nucifera*** | **10359** | **5389** | **1733** | **517** | **1450** | **140** |
| *Sorghum bicolor* | 11561 | 5383 | 1091 | 303 | 404 | 308 |
| *Zea mays* | 15116 | 10884 | 4603 | 260 | 2867 | 76 |
| *Oryza sativa* | 12660 | 8113 | 2929 | 143 | 1628 | 55 |
| *Phoenix dactylifera* | 10142 | 4964 | 2812 | 1033 | 716 | 307 |
| *Selaginella moellendorfii* | 8280 | 4055 | 1961 | . | 561 | . |
| 1 Solanaceae | 11230 | 4839 | 1652 | 110 | 628 | 86 |
| 2 Asterids | 9688 | 3969 | 300 | 64 | 346 | 44 |
| 3 Brassicaceae | 10118 | 4416 | 880 | 440 | 982 | 164 |
| 4 Brassicales | 9678 | 3496 | 82 | 226 | 61 | 214 |
| 5 Malvids | 9822 | 3666 | 75 | 86 | 38 | 113 |
| 8 Fabaceae | 10041 | 4990 | 492 | 179 | 1007 | 23 |
| 9 Strawberry+Legumes | 9728 | 3908 | 88 | 346 | 58 | 226 |
| 10 Fabids | 9986 | 4071 | 204 | 51 | 328 | 13 |
| 11 Eurosids | 9833 | 3734 | 283 | 73 | 144 | 70 |
| 12 Rosids | 9623 | 3634 | 213 | 42 | 140 | 135 |
| 13 Core-Eudicots | 9452 | 3620 | 365 | 56 | 233 | 83 |
| 14 Eudicots | 9143 | 3449 | 1129 | 47 | 491 | 74 |
| 15 Sorghum+Maize | 10773 | 4877 | 961 | 62 | 357 | 40 |
| 16 Poaceae | 9874 | 4479 | 1617 | 106 | 827 | 52 |
| 17 Monocots | 8363 | 3376 | 405 | 103 | 491 | 47 |
| 18 Monocots+Eudicots | 8061 | 2861 | 1742 | . | 592 | . |

**Table S9.** Maximum likelihood ancestral orthogroup reconstruction using a birth-death model that allows for lineage specific gain/loss rates with family-specific edge length variation with 1 category for the gamma distribution, as implemented in the program Count (Csurös 2010). Classification information for *Mimulus guttatus* and *Solanum lycopersicum* has been masked from this table to respect pre-publication data release restrictions.

| **TAXON/NODE** | **SINGLE** | **MULTI** | **GAIN** | **LOSS** | **EXPANSION** | **CONTRACTION** |
| --- | --- | --- | --- | --- | --- | --- |
| *Solanum tuberosum* | 5092 | 9190 | 3944 | 1215 | 3086 | 242 |
| *Arabidopsis thaliana* | 5377 | 5290 | 560 | 338 | 763 | 87 |
| *Thellungiella parvula* | 5573 | 5279 | 762 | 355 | 658 | 124 |
| *Carica papaya* | 7000 | 3991 | 2107 | 1290 | 715 | 462 |
| *Theobroma cacao* | 6576 | 6742 | 3554 | 432 | 1270 | 135 |
| *Populus trichocarpa* | 3786 | 8569 | 2433 | 342 | 4236 | 37 |
| *Fragaria vesca* | 6003 | 5248 | 2145 | 1166 | 1111 | 344 |
| *Medicago truncatula* | 4711 | 7688 | 4422 | 2303 | 1868 | 500 |
| *Glycine max* | 1938 | 9700 | 1941 | 583 | 5590 | 22 |
| *Vitis vinifera* | 6091 | 4317 | 1441 | 1147 | 1072 | 270 |
| ***Nelumbo nucifera*** | **4970** | **5389** | **1501** | **1028** | **2219** | **186** |
| *Sorghum bicolor* | 6178 | 5383 | 780 | 490 | 1022 | 205 |
| *Zea mays* | 4232 | 10884 | 4654 | 809 | 3640 | 180 |
| *Oryza sativa* | 4547 | 8113 | 2438 | 663 | 2459 | 179 |
| *Phoenix dactylifera* | 5178 | 4964 | 2612 | 1950 | 1743 | 352 |
| *Selaginella moellendorfii* | 4225 | 4055 | 1524 | 1188 | 2352 | 47 |
| 1 Solanaceae | 7543 | 4010 | 1530 | 204 | 811 | 127 |
| 2 Asterids | 7067 | 3160 | 200 | 72 | 210 | 28 |
| 3 Brassicaceae | 6153 | 4292 | 977 | 706 | 1300 | 140 |
| 4 Brassicales | 7153 | 3022 | 34 | 56 | 23 | 14 |
| 5 Malvids | 7185 | 3012 | 20 | 23 | 9 | 6 |
| 8 Fabaceae | 7195 | 3085 | 15 | 6 | 13 | 3 |
| 9 Strawberry+Legumes | 7197 | 3075 | 29 | 21 | 15 | 6 |
| 10 Fabids | 7199 | 3065 | 94 | 29 | 66 | 11 |
| 11 Eurosids | 7190 | 3009 | 136 | 52 | 51 | 18 |
| 12 Rosids | 7140 | 2975 | 30 | 14 | 11 | 6 |
| 13 Core-Eudicots | 7129 | 2970 | 300 | 87 | 187 | 39 |
| 14 Eudicots | 7071 | 2815 | 486 | 80 | 322 | 44 |
| 15 Sorghum+Maize | 6952 | 4319 | 476 | 89 | 289 | 66 |
| 16 Poaceae | 6798 | 4087 | 1808 | 403 | 1455 | 100 |
| 17 Monocots | 6960 | 2520 | 0 | 0 | 0 | 0 |
| 18 Monocots+Eudicots | 6960 | 2520 | 2171 | 636 | 1731 | 44 |

**Table S10.** Duplications in the lotus genome.

| **Multiplicity level** | **1** | **2** | **3** | **4** | **>4** | **All** |
| --- | --- | --- | --- | --- | --- | --- |
| # of ancestral loci | 5279 | 4289 | 279 | 165 | 80 | 10092 |
| # of genes | 5279 (19.8%) | 8578 (32.1%) | 837 (3.1%) | 660 (2.5%) | 510 (1.9%) | 15864 (59.4%) |
| Domain coverage (# of unique domains) | 2263 (1112) | 1861 (689) | 296 (20) | 174 (15) | 103 (1) | 3046 |

* Singleton homeologs in sacred lotus genome were compiled from intergenomic alignment with the grapevine and Arabidopsis genomes to be conservative in including sacred lotus specific genes.

**Table S11.** Phylogenetic timing of gamma duplications inferred from orthogroup phylogenetic histories. Bootstrap (BS)≥80 and BS≥50 are counts of nodes resolved with BS ≥80 or ≥50, respectively. Numbers shown in parentheses reflect additional resolved duplications following the inclusion of 34 MADS box unigenes (Vekemans et al., 2012) to five of the orthogroups.

|  | **Eudicot-wide** | | | **Core eudicot-wide** | | |
| --- | --- | --- | --- | --- | --- | --- |
|  | **BS≥80** | **BS≥50** | **BS≥0** | **BS≥80** | **BS≥50** | **BS≥0** |
| **Duplications** | 47 | 224 | 662 (663) | 69 | 195 (198) | 494 (498) |
| **Percent** | 40% | 53% | 57% | 60% | 47% | 43% |

**Table S12.** Conserved miRNA families in lotus.

| **microRNA family** | **No. of loci** | **No. of plant species with this family** |  |  |  |  |
| --- | --- | --- | --- | --- | --- | --- |
| miR156 | 7 | 31 |  | **continued** |  |  |
| miR159 | 1 | 25 |  | miR3627 | 3 | 1 |
| miR160 | 6 | 25 |  | miR3948 | 1 | 1 |
| miR162 | 1 | 18 |  | miR4414 | 1 | 2 |
| miR164 | 9 | 22 |  | **TOTAL** | **160** |  |
| miR165/166 | 10 | 27 |  |  |  |  |
| miR167 | 6 | 27 |  |  |  |  |
| miR168 | 4 | 22 |  |  |  |  |
| miR169 | 22 | 23 |  |  |  |  |
| miR171 | 12 | 28 |  |  |  |  |
| miR172 | 7 | 22 |  |  |  |  |
| miR319 | 5 | 24 |  |  |  |  |
| miR390 | 1 | 18 |  |  |  |  |
| miR393 | 4 | 15 |  |  |  |  |
| miR394 | 4 | 14 |  |  |  |  |
| miR395 | 10 | 20 |  |  |  |  |
| miR396 | 9 | 29 |  |  |  |  |
| miR397 | 1 | 17 |  |  |  |  |
| miR398 | 2 | 21 |  |  |  |  |
| miR399 | 11 | 21 |  |  |  |  |
| miR403 | 2 | 8 |  |  |  |  |
| miR408 | 2 | 21 |  |  |  |  |
| miR529 | 2 | 9 |  |  |  |  |
| miR535 | 1 | 8 |  |  |  |  |
| miR827 | 1 | 10 |  |  |  |  |
| miR845 | 1 | 3 |  |  |  |  |
| miR869 | 1 | 2 |  |  |  |  |
| miR1030 | 1 | 1 |  |  |  |  |
| miR1511 | 2 | 1 |  |  |  |  |
| miR1863 | 4 | 2 |  |  |  |  |
| miR2111 | 3 | 9 |  |  |  |  |
| miR2275 | 1 | 2 |  |  |  |  |
| miR2950 | 2 | 2 |  |  |  |  |
| TOTAL | 155 |  |  |  |  |  |

**Table S13.** Circadian clock associated genes in the lotus genome.

| ClockGene | Nn gene name | Nn ohnolog | RBB At gene | At gene name | Full At clock name |
| --- | --- | --- | --- | --- | --- |
| NnLHY | NNU_022090 |  | At1g01060 | LHY | LHY LATE ELONGATED HYPOCOTYL |
| NnRVE1 | NNU_025363 |  | At5g17300 | RVE1 | REVEILLE 1 |
| NnRVE4 | NNU_009563 |  | At5g02840 | RVE4 | REVEILLE 4 |
| NnRVE3A | NNU_020131 | NNU_010301 | NA |  | REVEILLE |
| NnRVE3B | NNU_010301 | NNU_020131 | NA |  | REVEILLE |
| NnRVE3C | NNU_020132 | NNU_020131 | NA |  | REVEILLE |
| NnLUX | NNU_007634 | NNU_011053 | At3g46640 | LUX/PCL1 | LUX ARRUTHMO, PHYTOCLOCK 1 |
| NnLVN | NNU_025377 |  |  |  |  |
| NnLUX4 | NNU_017077 |  | At3g10760 | LUX4 | LUX ARRUTHMO 4 |
| NnGI | NNU_010096 |  | At1g22770 | GI | GIGANTEA |
| NnCHEA | NNU_000404 | NNU_025322 | At5g08330 | CHE | CCA1 HIKING EXPEDITION/TCP |
| NnCHEB | NNU_025322 | NNU_000404 |  |  |  |
| NnSRR1 | NNU_025733 |  | At5g59560 | SRR1 | SENSITIVITY TO RED LIGHT REDUCED 1 |
| NnZTL | NNU_012826 |  | At5g57360 | ZTL/ADO1 | ZEITLUPE |
| NnFKF1 | NNU_000237 | NNU_016530 | At1g68050 | FKF1/ADO3 | FLAVIN-BINDING KELCH DOMAIN FBOX PROTEIN |
| NnFKF1B | NNU_016530 | NNU_000237 | NA |  |  |
| NnELF3A | NNU_024295 | NNU_013914 | At2g25930 | ELF3 | EARLY FLOWERING 3 |
| NnELF3B | NNU_013910 | NNU_024295 | NA |  |  |
| NnELF3C | NNU_013914 | NNU_024295 | NA |  |  |
| NnELF4A | NNU_005815 | NNU_011261 | At2g40080 | ELF4 | EARLY FLOWERING 4 |
| NnELF4E | NNU_011261 | NNU_005815 | NA |  |  |
| NnELF4C | NNU_010655 | NNU_018244 | At1g17455 | ELF4-L4 | EARLY FLOWERING 4-LIKE 4 |
| NnELF4D | NNU_018244 | NNU_010655 | NA |  |  |
| NnPRR1A | NNU_005424 | NNU_009558 | At5g61380 | PRR1/TOC1 | TIMING OF CAB1, PSEUDO-RESPONSE REGULATOR 1 |
| NnPRR1B | NNU_004828 |  | NA |  |  |
| NnPRR1C | NNU_009558 | NNU_005424 | NA |  |  |
| NnPRR3 | NNU_005578 | NNU_009558 | At5g60100 | PRR3 | PSEUDO-RESPONSE REGULATOR 3 |
| NnPRR9A | NNU_000512 |  | At2g46790 | PRR9 | PSEUDO-RESPONSE REGULATOR 9 |
| NnPRR9B | NNU_002600 | NNU_011459 | NA |  |  |
| NnPRR9C | NNU_011459 | NNU_002600 | NA |  |  |
| NnTEJ | NNU_020756 |  | At2g31870 | TEJ | SANSKRIT FOR 'BRIGHT' |
| NnTICA | NNU_007106 | NNU_001523 | At3g22380 | TIC | TIME FOR COFFEE |
| NnTICB | NNU_001523 | NNU_007106 | NA | TIC |  |
| NnXAP5 | NNU_017020 |  | At2g21150 | XPA5 |  |

**
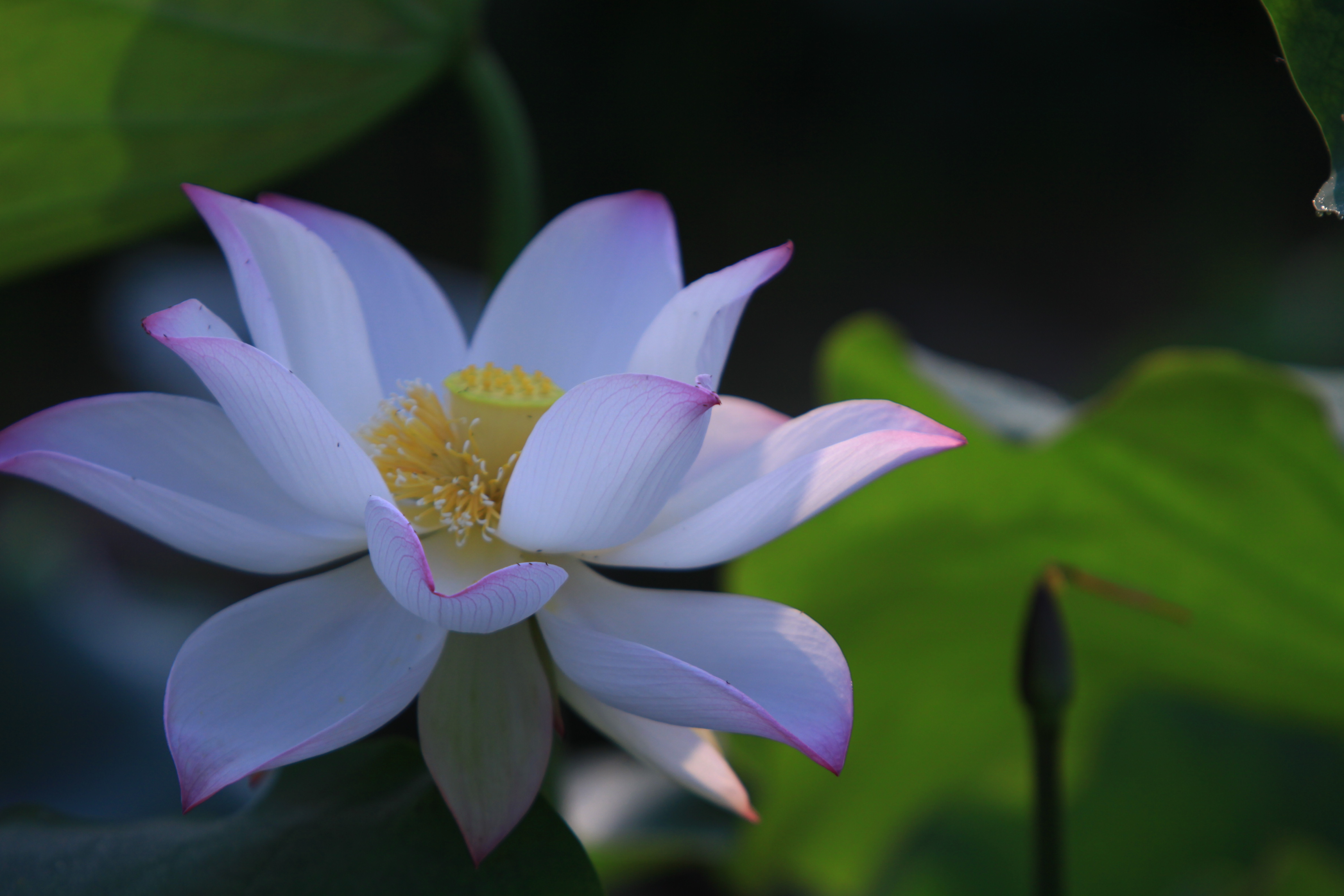
**

**A.**


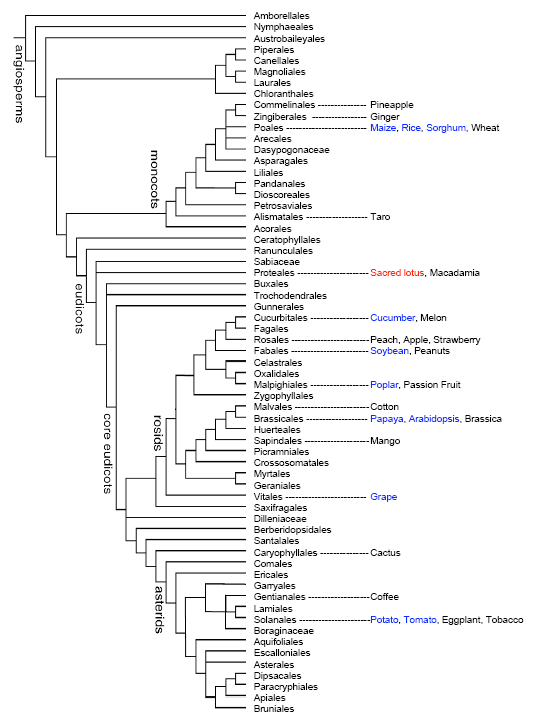


B

**Fig. S1.** Lotus flower morphology and phylogenetic interrelationship of flowering plants. (A) (A) Lotus flower (*Nelumbo nucifera* ), photo by Jianhong Zhan; (B) Selected species of agricultural importance are shown on the left and those in blue color have been sequenced. Tree was adapted from the Angiosperm Phylogeny Group [80].


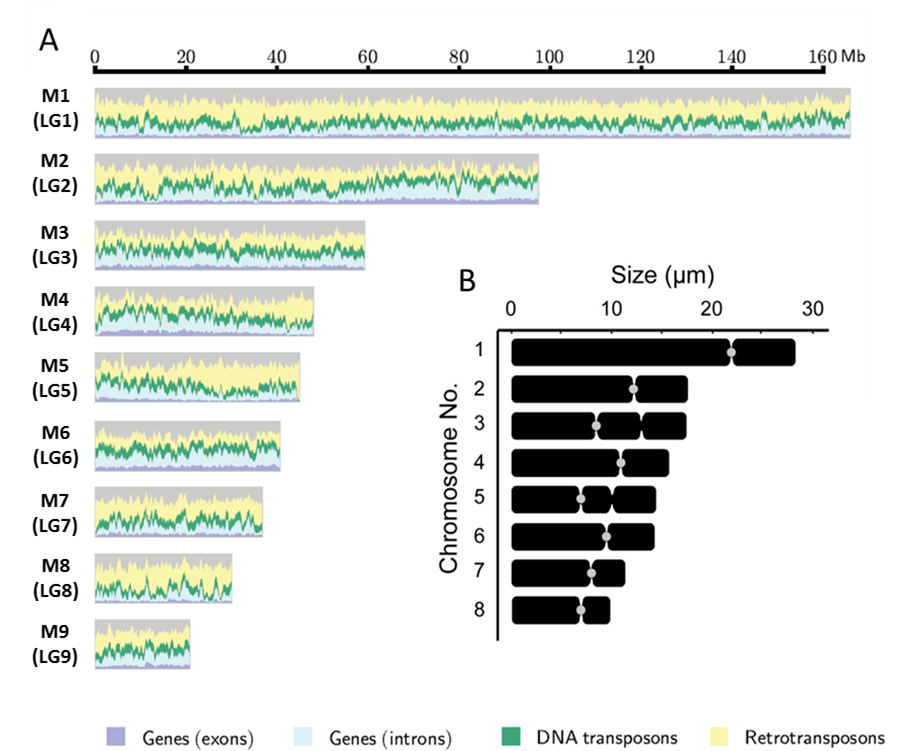


**Fig. S2.** Comparison of 9 anchored megascaffolds and lotus chromosome karyotype. (A) Major DNA components are classified into exons (blue), introns (cyan), DNA transposons (green), retrotransposons (yellow), with overall DNA contents of 6%, 18%, 16% and 32% of the genome sequence, respectively. The grey portion represents unclassified DNA contents. The plot was generated based on a moving window of 0.5Mb with 0.1Mb shift along each of the megascaffolds. (B) Illustration of chromosome karyotype of lotus cultivar "China Antique". Chromosome 3 and 5 have secondary constriction (usually rDNA cluster) and this might explain the less than proportional assembly of megascaffold 3.


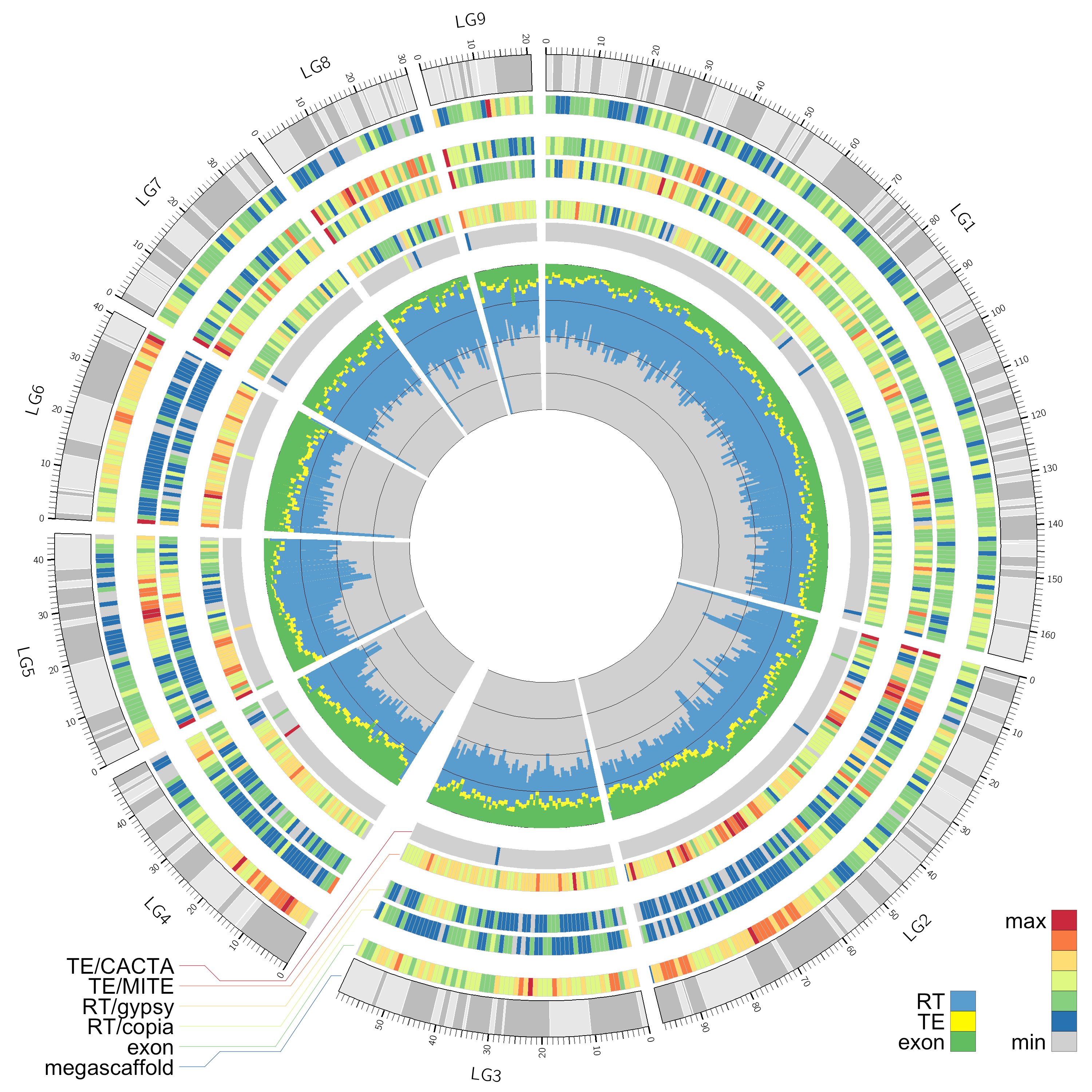


**Fig. S3.**  Sequence composition of the lotus assembly. The sequence composition is displayed as a Circos plot. The outermost ring displays the 9 anchored megascaffolds, with alternating dark and light bands to highlight the position of the individual scaffolds within, and tick marks every 1Mbp. The next 5 rings are heatmaps (grey-red) showing the density of exons, gypsy and copia retroposons, and CACTA and mite transposable elements. The innermost region shows the relative proportion of those sequence elements along the assembly. The axes rings in the innermost histogram represent 0%, 25%, 50%, 75%, and 100% for estimating the portion of each element.

**Fig. S4.** Expressed genes in rhizome, leaf, root and petiole tissues. 22,803 protein coding genes were expressed in 4 different tissues with 3,094 genes expressed in a tissue-specific manner.


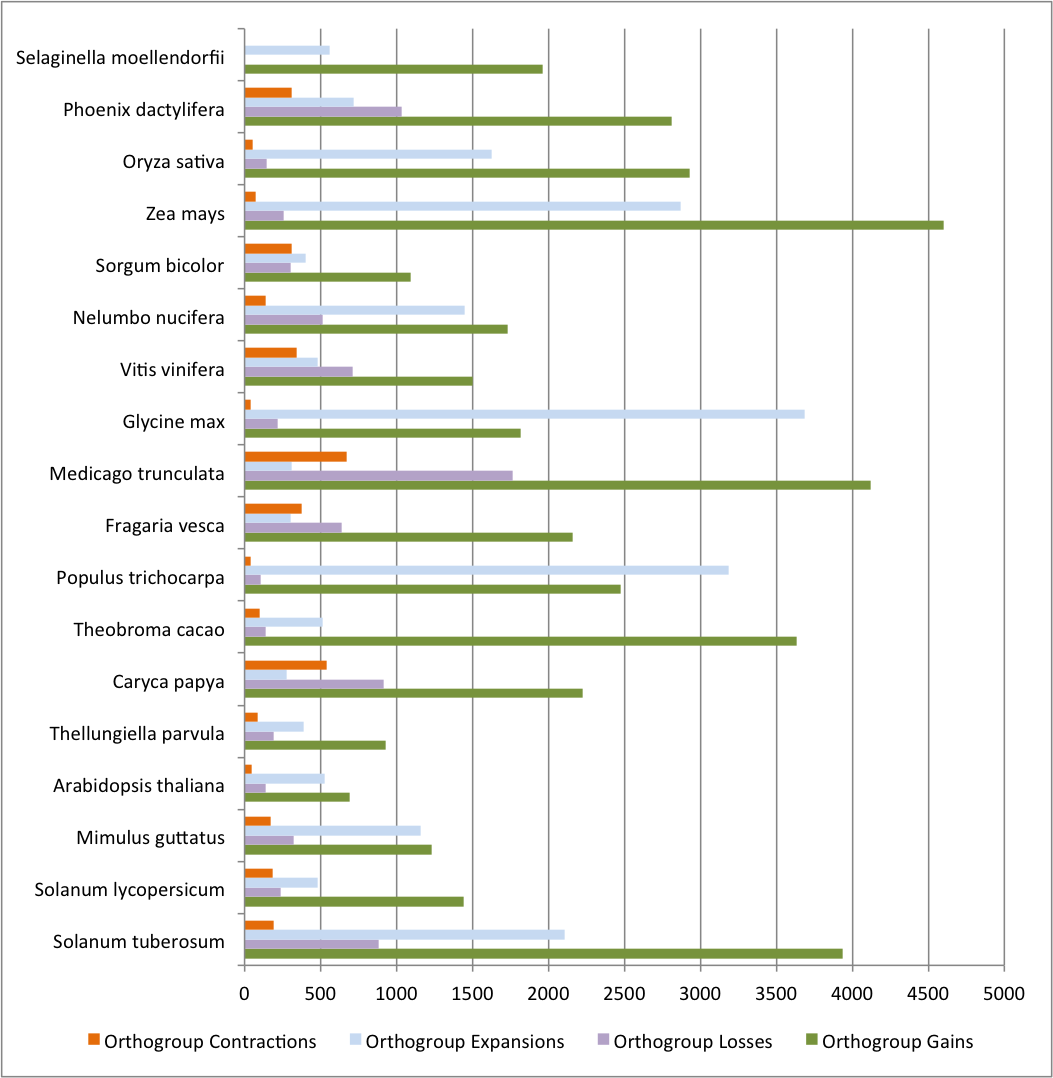


**Fig. S5.** Gains, losses, expansions, and contractions of gene families (orthogroups) in lotus and other sequenced angiosperm genomes.

**
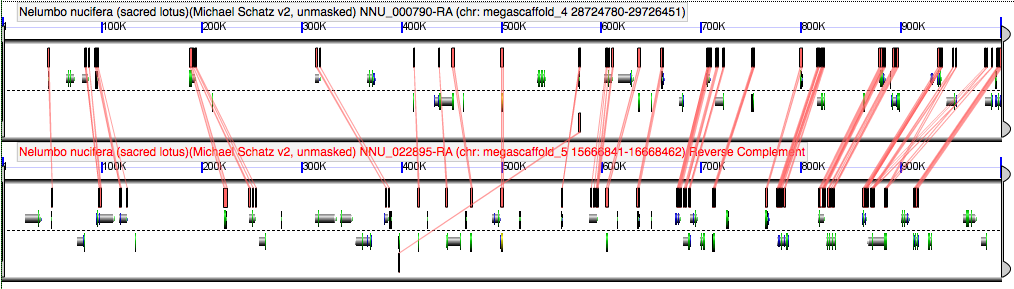
**

**Fig. S6.** High resolution analysis of intragenomic syntenic regions of lotus encompassing 1Mb of sequence from each region*.* Pink boxes and lines connect regions of sequence similarity for protein coding sequences. Note their collinear arrangement which is used to infer synteny. Results may be regenerated at <http://genomevolution.org/r/4pst>. Detailed explanation of the figure graphics and how to use GEvo are found at http://genomevolution.org/wiki/index.php?title=GEvo.


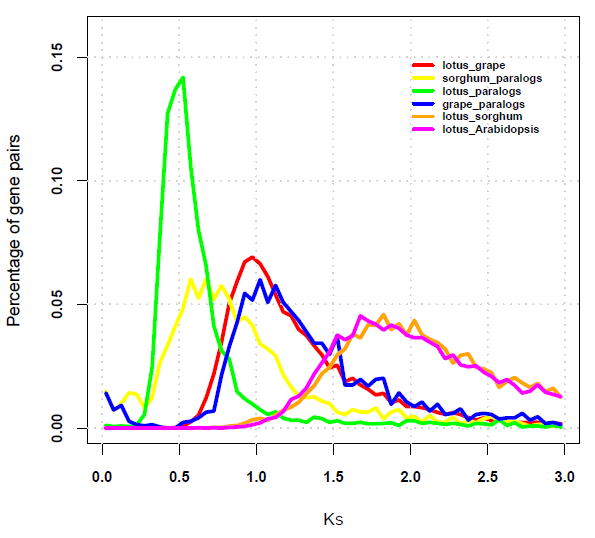


**Fig. S7.** Distribution of synonymous substitution rate (Ks) between homeologous gene pairs in intra- and inter- genomic comparisons.


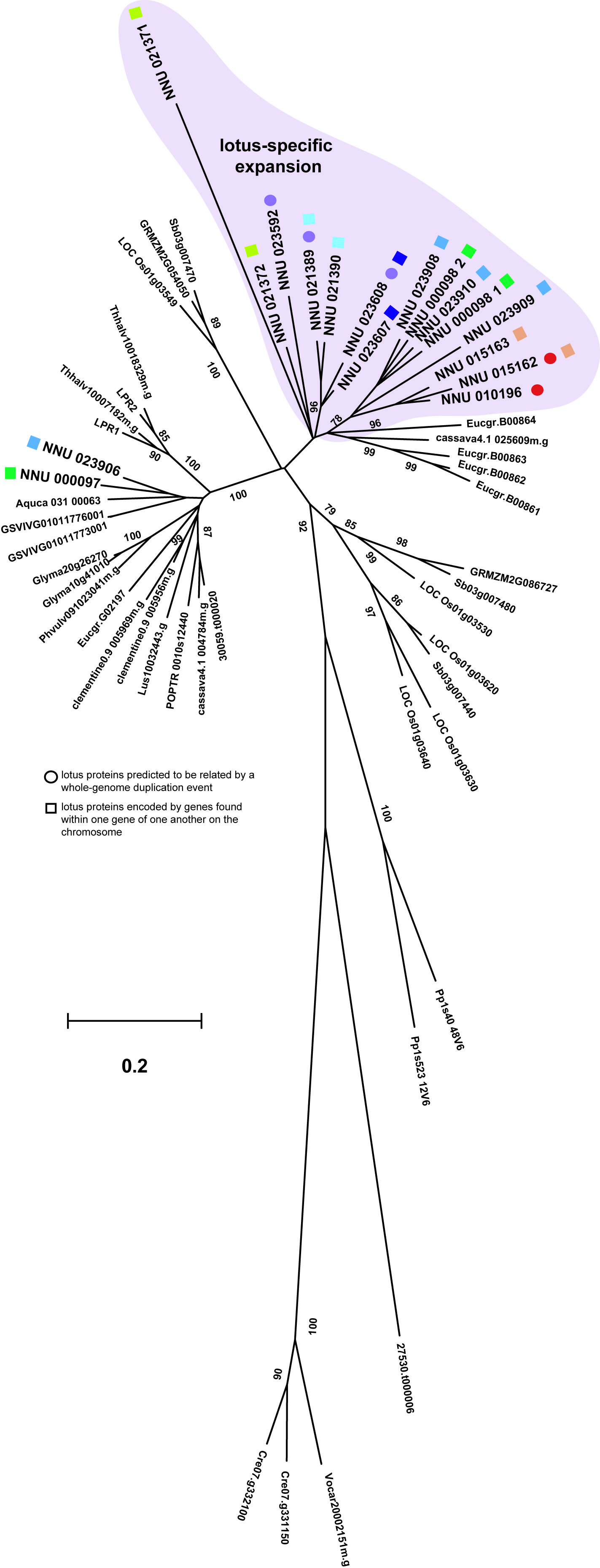


**Fig. S8.** Molecular Phylogenetic anaylsis of COG2132 members in the plant lineage

The evolutionary history was inferred by using the Maximum Likelihood method based on the JTT matrix-based model [96]. The bootstrap consensus tree inferred from 100 replicates [97] is taken to represent the evolutionary history of the protein sequences analyzed [97]. Branches corresponding to partitions reproduced in less than 50% bootstrap replicates are collapsed. Initial trees for the heuristic search were obtained automatically as follows. When the number of common sites was < 100 or less than one fourth of the total number of sites, the maximum parsimony method was used; otherwise BIONJ method with MCL distance matrix was used. The tree is drawn to scale, with branch lengths measured in the number of substitutions per site. The analysis involved 55 amino acid sequences. All positions containing gaps and missing data were eliminated. There were a total of 204 positions in the final dataset. Evolutionary analyses were conducted in MEGA5 [98]. Lotus protein sequences encoded by genes predicted to have arisen from a whole-genome duplication event are signified by the same color circle. Lotus proteins encoded by genes that are found in tandem on the genome are signified by the same color rectangle. Protein labels for *C. papaya, R. communis, V. vinifera, M. esculenta, P. patens,P. trichocarpa, C. clementina, S. bicolor, C. reinhardtii, V. carteri*, T*. halophila, Z. mays, O. sativa, A. coerulea, L. usitatissimum, P. vulgaris,* and *E. grandis* are from Phytozome (<http://www.phytozome.net/>). NNU_000098 contains two oxidase domains in tandem, which were split into independent sequences for the phylogenetic analysis (NNU_000098_1 and NNU_000098_2).


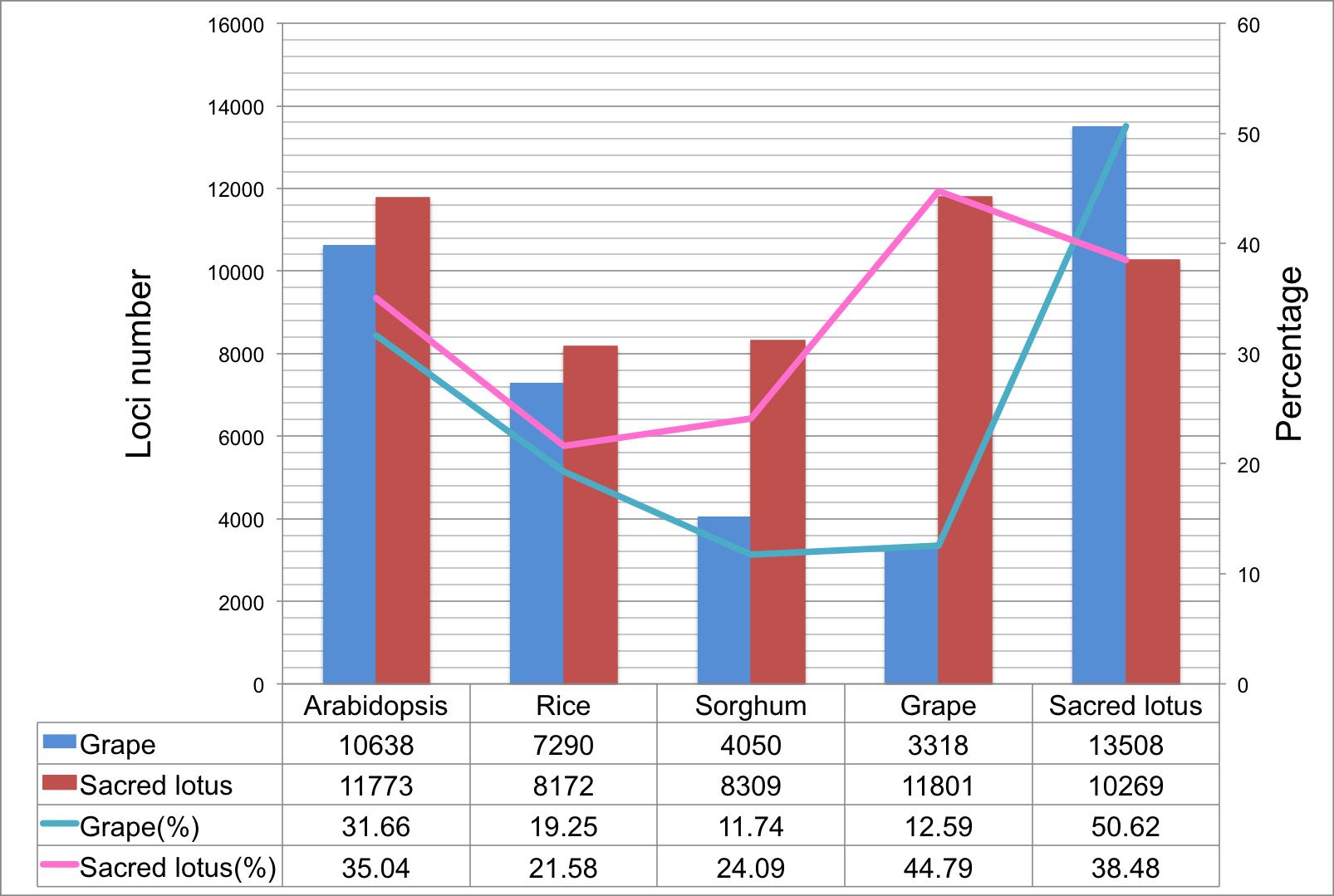


**Fig. S9.** Number and percentage of genes in the query genome having homeologous genes in the reference genome, using the grape or Sacred lotus genome as reference, and Arabidopsis, rice, and sorghum genomes as queries. All pairwise differences are statistically significant.

**Fig. S10.** Differences in mRNA length, CDS length, intron length, and percentage mRNA length difference attributable to intron length difference were measured for each pair of homeologous Sacred lotus genes. Extreme ranges on the X axes were trimmed for clarity.

**Fig. S11.** Ks distributions between orthologous homeologs gene pairs comparing the Sacred lotus genome to the grape genome (left) and sorghum genome (right). Red lines represent single copy homeologs in the Sacred lotus genome. The pairs of homeologs in the lotus genome are arbitrarily assigned to the high Ks value group (yellow lines) and low Ks group (green lines). If in all the duplets both Sacred lotus genes are equally distant from the grape/sorghum counterpart, it is expected the two sampling distributions should be alike.

**
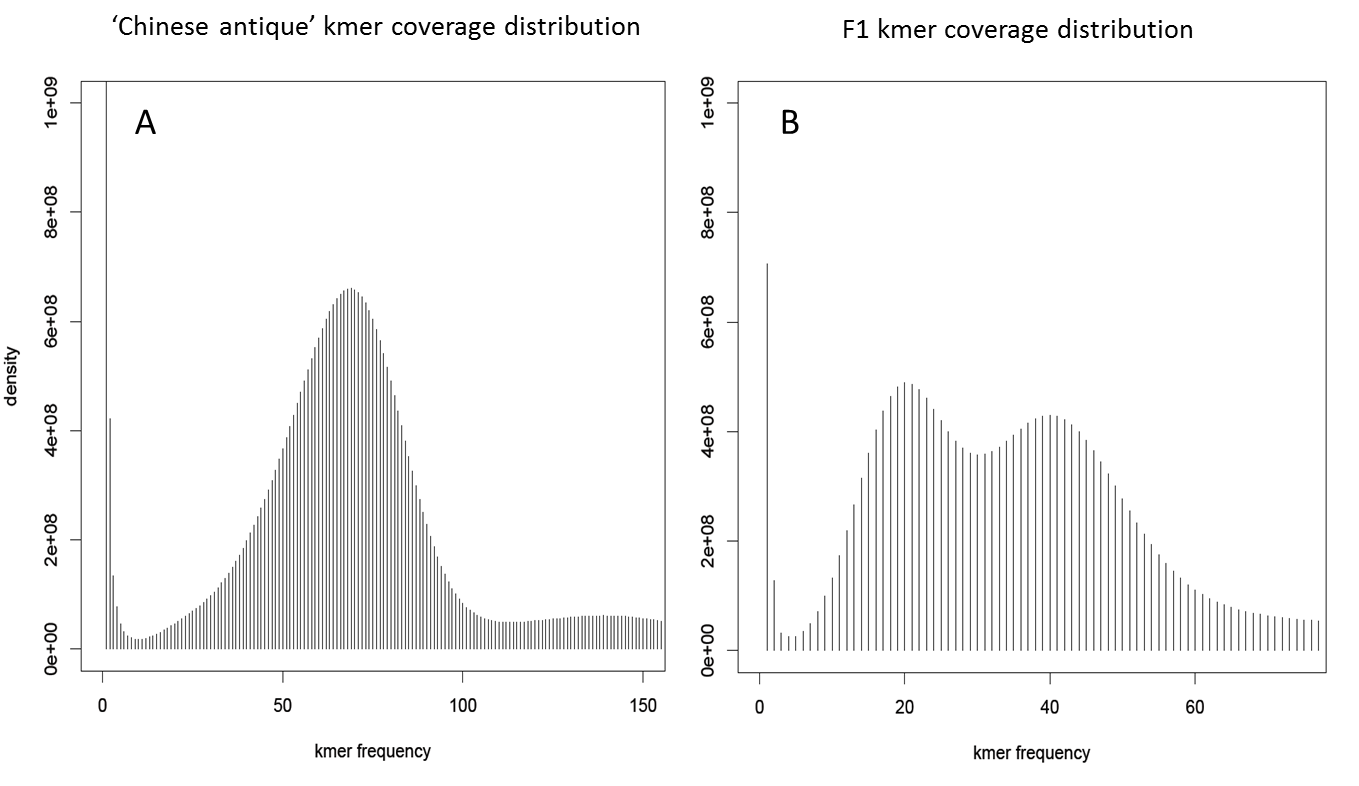
Fig. S12.** (A) Kmer distribution of unassembled ‘Chinese antique’ reads. (B) Kmer distribution of unassembled F1 ( N. nucifera ‘Chinese antique’ X *N. lutea*) reads. Coverage of ‘Chinese antique’ contigs displays a unimodal curve, whereas coverage of assembled contigs in the F1 shows a bimodal curve, indicating a low rate of heterozygosity in the ‘Chinese antique’ genome compared to the high rate in the F1 (as expected).


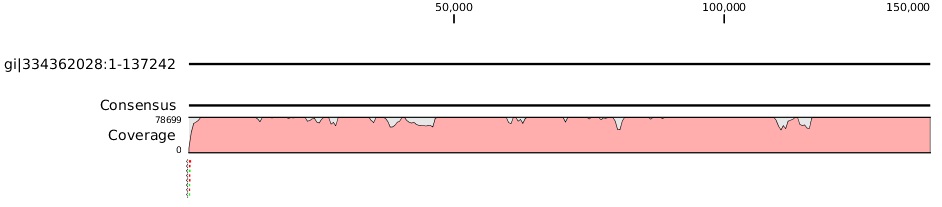


**Fig. S13.** Read coverage from lotus compared to the reference plastome (NC_015610). The solid top line represents the reference genome and the histogram shows the depth of reads ranging from 0 to 7869
